# Supplementary material for: Physical activity and sedentary behaviour interventions for people living with both frailty and multiple long-term conditions: a scoping review protocol
Source: BMJ Open. 2022 May 4;12(5):e061104. doi: 10.1136/bmjopen-2022-061104 (PMC9073409; doi:10.1136/bmjopen-2022-061104)
Supplement: Supplementary data [file bmjopen-2022-061104supp001.pdf]

Appendix 1. MEDLINE Search Strategy

1	exp comorbidity/
2	Comorbid*.mp.
3	co-morbid*.mp.
4	exp multimorbidity/
5	Multimorbid*.mp.
6	multi-morbid*.mp.
7	multidisease*.mp.
8	multi-disease*.mp.
9	exp multiple chronic conditions/
10	“multiple chronic conditions”.mp.
11	poly-morbidit*.mp.
12	polymorbidit*.mp.
13	poly-patholog*.mp.
14	polypatholog*.mp.
15	pluripatholog*.mp.
16	pluri-patholog*.mp.
17	multipatholog*.mp.
18	multi-patholog*.mp.
19	multicondition*.mp.

20	multi-condition*.mp.
21	exp Syndemic/
22	syndemic.mp.
23	"multiple health".mp.
24	"multiple chronic".mp.
25	"multiple long-term".mp.
26	condition*.mp.
27	ill*.mp.
28	disorder*.mp.
29	disease*.mp.
30	23 or 24 or 25
31	26 or 27 or 28 or 29
32	30 and 31
33	exp Diabetes Mellitus, Type 2/
34	“diabetes type 2”.mp.
35	“diabetes type II”.mp.
36	“early onset diabetes”.mp.
37	(diabetes adj3 type 2).mp.
38	(diabetes adj2 type ii).mp.
39	(diabetes adj type two).mp.

40	T2DM.mp.
41	T2D.mp.
42	33 or 34 or 35 or 36 or 37 or 38 or 39 or 40 or 41
43	exp Hypertension/
44	exp Blood Pressure/
45	“Blood Pressure”.mp.
46	hypertens*.mp.
47	43 or 44 or 45 or 46
48	exp Heart Diseases/
49	exp Heart Failure/
50	exp Cardiovascular Diseases/
51	exp Coronary Disease/
52	cardiac.mp.
53	heart.mp.
54	cardiovascular.mp.
55	coronary.mp.
56	52 or 53 or 54 or 55
57	31 and 56
58	48 or 49 or 50 or 51 or 57
59	exp Vascular Diseases/

60	exp Carotid Artery Diseases/
61	cerebrovascular.mp.
62	vascular.mp.
63	Carotoid*.mp.
64	Arter*.mp.
65	61 or 62 or 63 or 64
66	31 and 65
67	exp Cerebrovascular Disorders/
68	59 or 60 or 66 or 67
69	exp Asthma/
70	asthma*.mp.
71	69 or 70
72	exp Pulmonary Disease, Chronic Obstructive/
73	COPD.mp.
74	“chronic obstructive pulmonary disease”.mp.
75	72 or 73 or 74
76	exp Hyperlipidemias/
77	exp Hypercholesterolemia/
78	exp Hypertriglyceridemia/
79	hyperlipid*em*.mp.

80	hypercholesterol*emia$.mp.
81	hypertriglycerid*emia*.mp.
82	76 or 77 or 78 or 79 or 80 or 81
83	exp Arthritis/
84	exp Arthritis, Rheumatoid/
85	exp Osteoarthritis/
86	arthritis.mp.
87	“rheumatoid arthritis”.mp.
88	osteoarthritis.mp.
89	83 or 84 or 85 or 86 or 87 or 88
90	exp Depression/
91	exp Anxiety/
92	depression.mp.
93	anxiety.mp.
94	90 or 91 or 92 or 93
95	exp Neoplasms/
96	malignan*.mp.
97	cancer.mp.
98	95 or 96 or 97
99	exp HIV/

100	exp Acquired Immunodeficiency Syndrome/
101	"acquired immunodeficiency syndrome".mp.
102	AIDS.mp.
103	HIV.mp.
104	99 or 100 or 101 or 102 or 103
105	exp Kidney Failure, Chronic/
106	exp Kidney Diseases/
107	exp Renal Insufficiency/
108	renal.mp.
109	kidney.mp.
110	108 or 109
111	31 and 110
112	105 or 106 or 107 or 111
113	exp Liver Diseases/
114	liver.mp.
115	31 and 114
116	113 or 115
117	exp Osteoporosis/
118	osteoporosis.mp.
119	117 or 118

120	exp Obesity/
121	obes*.mp.
122	120 or 121
123	exp Multiple Sclerosis/
124	multiple sclerosis.mp.
125	"MS".mp.
126	123 or 124 or 125
127	exp Parkinson Disease/
128	“parkinsons disease”.mp.
129	"PD".mp.
130	127 or 128 or 129
131	peripheral artery disease.mp.
132	exp Peripheral Arterial Disease/
133	131 or 132
134	co-occur*.mp.
135	cooccur*.mp.
136	concurrent.mp.
137	coexist*.mp.
138	co-exist*.mp.
139	134 or 135 or 136 or 137 or 138

140	31 and 139
141	1 or 2 or 3 or 4 or 5 or 6 or 7 or 8 or 9 or 10 or 11 or 12 or 13 or 14 or 15 or 16 or 17 or 18 or 19 or 20 or 21 or 22 or 32
142	42 or 47 or 58 or 68 or 71 or 75 or 82 or 89 or 94 or 98 or 104 or 112 or 116 or 119 or 122 or 126 or 130 or 133
143	140 and 142
144	141 or 143
145	exp Frail Elderly/
146	exp Frailty/
147	frail*.mp.
148	prefrail.mp.
149	pre-frail.mp.
150	“functionally-impaired”.mp.
151	Physical function*.mp.
152	"Physical frail*".mp.
153	Debility.mp.
154	145 or 146 or 147 or 148 or 149 or 150 or 151 or 152 or 153
155	exp Exercise/
156	exp Physical Fitness/
157	exp Sedentary Behavior/

158	exp Rehabilitation/
159	exp Exercise Therapy/
160	exercis*.mp.
161	“physical activit*.mp.
162	physical fitness.mp.
163	sedentary behavio*r”.mp.
164	sedentary.mp.
165	rehabilitat*.mp.
166	“exercise therapy”.mp.
167	155 or 156 or 157 or 158 or 159 or 160 or 161 or 162 or 163 or 164 or 165 or 166
168	144 and 154 and 167
169	limit 168 to yr="2000 -Current"
