# Supplementary material for: Physical activity and sedentary behaviour interventions for people living with both frailty and multiple long-term conditions: a scoping review protocol
Source: BMJ Open. 2022 May 4;12(5):e061104. doi: 10.1136/bmjopen-2022-061104 (PMC9073409; doi:10.1136/bmjopen-2022-061104)
Supplement: Author's manuscript [file bmjopen-2022-061104.draft_revisions.pdf]

BMJ Open is committed to open peer review. As part of this commitment we make the peer review history of every article we publish publicly available.

When an article is published we post the peer reviewers' comments and the authors' responses online. We also post the versions of the paper that were used during peer review. These are the versions that the peer review comments apply to.

The versions of the paper that follow are the versions that were submitted during the peer review process. They are not the versions of record or the final published versions. They should not be cited or distributed as the published version of this manuscript.

BMJ Open is an open access journal and the full, final, typeset and author-corrected version of record of the manuscript is available on our site with no access controls, subscription charges or pay-per-view fees (http://bmjopen.bmj.com).

If you have any questions on BMJ Open's open peer review process please email info.bmjopen@bmj.com

BMJ Open

Physical activity and sedentary behaviour interventions for people living with both frailty and multiple long-term conditions: a scoping review protocol

Journal:	*BMJ Open*
Manuscript ID	bmjopen-2022-061104
Article Type:	Protocol
Date Submitted by the Author:	17-Jan-2022
Complete List of Authors:	Young, Hannah; University Hospitals of Leicester NHS Trust, Leicester Diabetes Centre, College of Life Sciences; University of Leicester, Diabetes Research Centre<br>Yates, Thomas; University of Leicester, Diabetes Research Centre, College of Life Sciences<br>Dempsey, Paddy ; University of Leicester, Diabetes Research Centre, College of Life Sciences; Cambridge Biomedical Campus, MRC Epidemiology Unit, Institute of Metabolic Science<br>Herring, Louisa ; University Hospitals of Leicester NHS Trust, Leicester Diabetes Centre,<br>Henson, Joseph; University Hospitals of Leicester NHS Trust, Leicester Diabetes Centre; University of Leicester, Diabetes Research Centre, College of Life Sciences,<br>Sargeant, Jack; University Hospitals of Leicester NHS Trust, Leicester Diabetes Centre; University of Leicester, Diabetes Research Centre, College of Life Sciences,<br>Curtis, Ffion; University Hospitals of Leicester NHS Trust, NIHR Applied Research Collaboration East Midlands<br>Sathanapally, Harini; University Hospitals of Leicester NHS Trust, NIHR Applied Research Collaboration East Midlands<br>Highton, Patrick; University Hospitals of Leicester NHS Trust, NIHR Applied Research Collaboration East Midlands<br>Hadjiconstantinou, Michelle; University of Leicester, Diabetes Research Centre, College of Life Sciences<br>Pritchard, Rebecca; University Hospitals of Leicester NHS Trust, NIHR Leicester BRC; University of Edinburgh<br>Lock, Selina; University of Leicester, Library Research Services<br>Singh, Sally; University Hospitals of Leicester NHS Trust, 12. Department of Respiratory Medicine; Leicester Biomedical Research Unit, Centre for Exercise and Rehabilitation Science<br>Davies, Melanie; University of Leicester, Diabetes Research Centre; University Hospitals of Leicester NHS Trust, Leicester Diabetes Centre
Keywords:	Rehabilitation medicine < INTERNAL MEDICINE, REHABILITATION MEDICINE, SPORTS MEDICINE

SCHOLARONE™
Manuscripts

Physical activity and sedentary behaviour interventions for people living with both frailty and multiple long-term conditions: a scoping review protocol

Hannah ML Young[1,2,3,], Thomas Yates[2], Paddy C Dempsey[2,4,5], Louisa Y Herring[1], Joseph Henson[1,2], Jack Sargeant[1,2], Ffion Curtis[6], Harini Sathanapally[6], Patrick Highton[1,6], Michelle Hadjiconstantinou[2], Rebecca Pritchard[1,7,9], Selina Lock[10], Sally J Singh[11,12], Melanie Davies[1,2]

1. Leicester Diabetes Centre, University of Hospitals of Leicester NHS Trust, Leicester, UK.
2. Diabetes Research Centre, College of Life Sciences, University of Leicester, UK.
3. Department of Respiratory Sciences, College of Life Sciences, University of Leicester, UK.
4. MRC Epidemiology Unit, Institute of Metabolic Science, University of Cambridge, Cambridge Biomedical Campus, Cambridge, UK.
5. Baker Heart and Diabetes Institute, Melbourne, Australia.
6. NIHR Applied Research Collaboration East Midlands, Leicester General Hospital, Leicester, UK.
7. University of Edinburgh, Edinburgh, UK
8. University Hospitals of Leicester NHS Trust, Leicester, UK
9. NIHR Leicester BRC, Cardiovascular Research Centre, Glenfield Hospital, Leicester, UK
10. Library Research Services, University of Leicester, Leicester, UK.
11. Centre for Exercise and Rehabilitation Science, Leicester Biomedical Research Unit, Leicester, UK.
12. Department of Respiratory Medicine, Glenfield Hospital, University Hospitals of Leicester NHS Trust, Leicester, UK.

Corresponding author:

Hannah Young

Leicester Diabetes Centre

Leicester General Hospital

Gwendolen Road

For peer review only

For peer review only - http://bmjopen.bmj.com/site/about/guidelines.xhtml

Leicester

UK

LE4 5PW

hy162@le.ac.uk

Abstract word count: 234

Main text word count: 3827

Keywords: multimorbidity, frailty, physical activity, exercise, review, multiple conditions, sedentary behaviour

For peer review only

For peer review only - http://bmjopen.bmj.com/site/about/guidelines.xhtml

Abstract

Introduction

The number of people living with multiple long-term conditions (MLTC) is predicted to rise. Within this population, those also living with frailty are particularly vulnerable to poor outcomes, including decreased function. Increased physical activity, including exercise, has the potential to improve function in those living with both MLTCs and frailty but to date, the focus has remained upon older people and may not reflect outcomes for the growing number younger people living with MLTC and frailty. For those with higher burdens of frailty and MLTCs, physical activity may be challenging. Tailoring physical activity in response to symptoms and periods of ill-health, involving family, and reducing sedentary behaviour may be important in this population. How the tailoring of interventions has been approached within existing studies is currently unclear. This scoping review aims to map the available evidence regarding these interventions in people living with both frailty and MLTCs.

Methods and analysis

We will use a six-stage process: (1) identifying the research questions; (2) identifying relevant studies (via database searches); (3) selecting studies; (4) charting the data; (5) collating and summarising and (6) stakeholder consultation. Studies will be critically appraised using the Mixed Methods Appraisal Tool.

Ethics and dissemination

All data in this project will be gathered through database searches. Stakeholder consultation will be undertaken with an established patient and public involvement group. We will disseminate our findings via social media, publication, and engagement meetings.

For peer review only - http://bmjopen.bmj.com/site/about/guidelines.xhtml

Strengths and limitations of this study

- This review will update and extend existing reviews by populations of all ages living with frailty and multiple long-term conditions (MLTCs), which reflects the changing demographic of the population.
- We will identify primary studies that have incorporated sedentary behaviour, physical activity and/or exercise interventions and report how they were modified and tailored to the needs of those with MLTCs and frailty.
- The design of the review has been shaped by strong patient and public involvement, and a diverse range of stakeholders will be involved throughout the review.
- The exclusion of non-English language studies may represent a limitation.
- There is potential that our review may miss some relevant studies, but an initial search indicates a need to maintain a balance between reviewing a feasible number of studies and ensuring all relevant articles are included.

For peer review only

For peer review only - http://bmjopen.bmj.com/site/about/guidelines.xhtml

Introduction

Physical inactivity and sedentary behaviour in people with multiple long-term conditions and frailty

The number of people living with multiple long-term conditions (MLTCs), defined as the coexistence of two or more chronic conditions, is a growing global health concern. [1] The presence of MLTC is associated with a myriad of poor outcomes, including low physical function and a resulting loss of independence and life participation.[2,3,4,5,6,7,8,9] Within this population, those also living with frailty, defined as "a multidimensional syndrome of decreased physiological reserve leading to increased vulnerability to minor health stressors", represent a group who are particularly vulnerable to these poor outcomes.[10,11] This is, in part, due to a bi-directional relationship between frailty and MLTCs.[12,13] Frailty appears to contribute to the onset of MLTCs, whilst the presence of MLTCs increases the risk of developing frailty.[14,15] Evidence suggests that people with MLTCs are at risk of becoming frail earlier.[16,17]

Sedentary behaviour (defined as any activity performed in a seated or reclined posture that requires low energy expenditure) and physical inactivity (insufficient amounts of moderate-to-vigorous physical activity) are hallmarks of many long-term conditions.[18,19,20] Reducing physical inactivity and encouraging people to participate in exercise are often primary targets for intervention, but there is also growing interest in reducing sedentary behaviour.[21] Physical inactivity is common within the context of MLTCs. Approximately 29.2% of people living with MLTCs are unable to achieve physical activity recommendations, with levels of inactivity increasing with age.[22] In addition, there is an increased prevalence of probable sarcopenia in people with MLTCs.[23] Within this group there is a graded relationship between the degree of multimorbidity and sarcopenia, and an independent association between sarcopenia and physical inactivity.[24] People with MLTCs who can exercise demonstrate improved outcomes, indicating that physical activity and exercise may be important in the management of MLTCs.[20,21,25,26,27,28,29]

For peer review only - http://bmjopen.bmj.com/site/about/guidelines.xhtml

Sarcopenia, physical inactivity, and sedentary behaviour are also modifiable characteristics of the frailty phenotype and existing evidence suggests that undertaking resistance exercise and physical activity may delay both the onset and progression of frailty and improve outcomes.[30,31,32,33,34,35] Increased physical activity is also a cornerstone of obesity management, which has also been associated with MLTCs and frailty.[36,37,38] Reducing sedentary behaviour and engaging in physical activity and exercise, particularly resistance training, across the life-course, and when living with MLTC, is now a major focus of clinical and public health policy.[21,32,39,40,41]

Compared to data from 2015, an 86% increase in the number of people living with two more conditions is predicted by 2035, in those aged over 65 years alone, making effective interventions for this groups increasingly important.[42,43] This is underscored by a recent Public Health England report which underlines the negative impact of the COVID-19 pandemic on sedentary behaviour and physical activity, particularly upon those with MLTCs who were advised to shield. Modelling predicts that the impact of deconditioning and increased falls risk will lead an estimated cost of £211 million per year to health and social care services.[44]

Sedentary behaviour and physical activity and interventions

Existing systematic reviews indicate that structured exercise can lead to improvements in function, depression, and anxiety, but have mixed impacts upon quality of life in people living with MLTCs who are not frail.[45,46,47] Similarly, systematic reviews of exercise in people living with frailty suggest that those which include resistance training may improve function but have an unclear impact upon quality of life or ability to participate in activities of daily living.[48,49] Only one of these reviews, published almost ten years ago, has examined the effectiveness of exercise for people living with both MLTCs and frailty, but focuses specifically on older people (aged over 60 years), and is limited by the interchangeable use of the concepts of disability and frailty.[45] Contemporary research indicates that the demographics of people living with frailty and MLTCs are changing, with accelerated rates of both phenomena being observed in younger populations, people living with obesity, those with new and emerging health issues such as long-term HIV and early onset Type II

For peer review only - http://bmjopen.bmj.com/site/about/guidelines.xhtml

diabetes.[15,36,50,51] An updated review of sedentary behaviour and physical activity interventions for those living with both MLTCs and frailty, that includes these groups is clearly warranted.[52]

Existing evidence suggests that identifying and targeting non-disease specific issues (for example symptoms such as pain, fatigue breathlessness) are important factors associated with increased effectiveness.[47,53] This may be important within the context of physical activity and exercise interventions, where uptake is hampered by concerns that symptoms may be aggravated.[54] Additionally, adherence in this population is low and the sustained engagement of people with both MLTCs and frailty is disrupted by periods of ill-health, exacerbation of their conditions and increased symptom burden, coupled with lack of guidance and support on safe and effective physical activity in these circumstances.[36,55,56,57]

Guidance on the management of people living with MLTCs and frailty highlights a need to identify and tailor treatment strategies.[41] Tailored adaptations to physical activity and exercise may encourage people living with frailty and MLTC to engage and support them to continue as much as they are safely able during periods of ill-health, increased symptomology and functional variability. Such strategies are urgently needed, as those who do complete combined resistance and aerobic exercise programmes appear to benefit from a range of improved outcomes, including improved exercise performance, physical activity levels and health status.[55] Whilst recently published guidance supports healthcare professionals to address concerns relating the exacerbation of common symptoms at the point of initiating physical activity and exercise, they do not address how such interventions might need to be modified dependent upon symptom severity, which appears to be important for sustainability.[54] Even less is known about the role of interventions to address sedentary behaviour in this population.[19,53,58,59,60] Reducing sedentary behaviour may form a particularly useful adjunct approach for those who lack the capacity or motivation to undertake physical activity and exercise. For these individuals, reducing and breaking up sedentary time may offer a more realistic, acceptable starting point, and a more sustainable segue into exercise.[61,62]

For peer review only - http://bmjopen.bmj.com/site/about/guidelines.xhtml

Finally, contextual factors, such as family involvement have been cited as key factors which may influence engagement and consequently outcomes.[47] Indeed, qualitative research indicates that family members and significant others are important 'gatekeepers' to health services and independence in people living with MLTCS and frailty.[58,63] This suggests that family involvement may be an important component of an intervention. Evidence also suggests that MLTCs may occur within families beyond parent/child dyads, particularly within people from minority ethnic backgrounds.[64,65,66,67] These are associated with increased sedentary behaviour, physical inactivity, and poor levels of function at the family level.[64,65,66,67,68,69] Therefore, in addition to improving the engagement of those living with MLTCS and frailty, the involvement of family members may help to reduce their risk of developing MLTCs or support them to manage existing LTCs. Indeed, the findings of a recent systematic review suggest that the engagement of carers in an exercise 'dyad' leads to improvements in both caregiver physical and psychosocial health.[70] The involvement of carers in sedentary behaviour and physical activity interventions is less clear. Caregiving requirements are likely to vary by age, and as a result of different clusters of MLTCs, together with frailty, so different caregivers and family members may need to be involved in different ways. Clarifying how carers and family members have been involved in sedentary behaviour, physical activity and exercise interventions for people with MLTCs and frailty may help identify further ways to tailor such interventions and leverage intergenerational support.

Overall, understanding the characteristics of sedentary behaviour, physical activity and exercise interventions for people living with both MLTCs and frailty is an important first step to identifying safe, scalable, and sustainable physical activity interventions which may be beneficial in this population.

Justification for this review

Scoping reviews seek to 'determine the body of literature on a given topic and give a clear indication of the volume of literature available, as well as an overview of its focus'.[71] Whilst

For peer review only - http://bmjopen.bmj.com/site/about/guidelines.xhtml

there is emerging evidence in the contexts of MLTCs and frailty independently, less is known about the role of sedentary behaviour and physical activity interventions in those living with both. Specifically, we would like to identify primary studies in these conditions, including younger adults where very little research has been conducted. Conducting a scoping review will enable us to draw on the wider literature and begin to develop a programme theory to underpin our intervention design targeting this increasing population.[72,73,74,75,76] Of relevance to this population is scoping the range of ways in which interventions may be adapted and tailored. An approach that allows diverse evidence to be integrated will provide a more nuanced understanding of this, which may be lost within a systematic review.[76,77]

Aim and objectives

The aim of this review is to map the available evidence on the use of sedentary behaviour, physical activity and exercise interventions in people living with both frailty and MLTCs. The identified evidence will be used to highlight gaps within the existing literature, including a determination of whether there is sufficient evidence in this area to undertake a systematic review.[76] The results of the review will be used to inform the design and development of an intervention for this population.[73]

<u>Methods and analysis</u>

Design

This scoping review will follow a six-stage process informed by guidance from Arksey and O'Malley (2005),[78] and subsequent refinements outlined by Levac (2010),[79] Colquhorn (2014)[80] and Daudt (2013).[81] This protocol follows the PRISMA reporting guidelines for scoping reviews.[77]

1.Identifying the research questions

To achieve the above aims the following questions will be addressed:

For peer review only - http://bmjopen.bmj.com/site/about/guidelines.xhtml

What are the characteristics of sedentary behaviour, physical activity and exercise interventions that have been used with people living with both frailty and MLTCs?

How have carers and relatives been included within the design, development, and delivery of these interventions?

For each of the above, what approaches appear to contribute to improved engagement and outcomes, particularly physical function?

Patient and public representatives have been involved in shaping these questions from the outset. They described struggling to maintain physical activity levels during periods of ill-health, compounded by barriers to accessing existing services which could not accommodate their needs, or their variable ability to engage due to symptoms.

2. Identifying relevant studies

Inclusion and exclusion criteria

All relevant literature will be included, irrespective of the study design utilised. Studies will be included if the meet the following inclusion criteria (summarised within Table 1). These parameters may be refined and adapted if unmanageable volumes of eligible studies are identified following an initial search.[78]

For peer review only - http://bmjopen.bmj.com/site/about/guidelines.xhtml

Table 1. Inclusion and exclusion criteria

Inclusion criteria	Exclusion criteria
Any study design examining interventions or intervention content (e.g. single-group preintervention and postintervention, parallel- group design crossover or cluster, qualitative studies and process evaluations relating to interventions)	Studies in children or animals
Adults aged 18 and above	Presence of MLTCs not defined or < 50% of the sample report MLTCs
Living with both frailty and MLTCs	Recognised measure of frailty or validated proxy not used
Interventions with a sedentary behaviour, physical activity or exercise focus, including multicomponent interventions	Non-English language studies
Any setting	Studies of acute responses to sedentary behaviour, physical activity, or exercise, including interventions of less than one week in duration

Population

Adults aged 18 or above, living with both MLTCs and frailty. Within the review we define MLTCs as the co-existence of two or more chronic conditions (physical or mental) in a single individual.[1] Included long-term conditions, which could feasibly be influenced by physical activity, are outlined within Table 2. Studies where the presence of MLTCs could be assumed based solely upon participants' age or circumstances (e.g., resident of a nursing home) will be excluded.[53] However, given the relatively recent use of MLTCs as a term, we will include studies where the characteristics of the sample indicate that the majority (over 50%) are living

For peer review only - http://bmjopen.bmj.com/site/about/guidelines.xhtml

with MLTCs (e.g., Charlson co-morbidity index scores, counts of conditions). Similar approaches have been adopted within previous reviews of exercise and MLTCs.[46]

Table 2 Conditions included within the review

Type 2 diabetes	Asthma
COPD	Arthritis (osteo and rheumatoid)
Depression	Anxiety
Cancer (solid organ, haematological and metastatic)	HIV and AIDS
Chronic kidney disease	Chronic liver disease
Heart failure	Peripheral artery disease
Coronary artery disease	Hyperlipidaemia
Obesity	Ischaemic heart disease
Osteoporosis	Multiple Sclerosis
Parkinsons disease	

We define frailty as "a multidimensional syndrome of decreased physiological reserve leading to increased vulnerability to minor health stressors".[10] Studies using a validated frailty measure (e.g., Fried Frailty Phenotype, Clinical Frailty Scale, Frailty Index, Electronic Frailty Index) will be prioritised. Considering that frailty in younger people with MLTCs has only recently begun to be recognised, we will also include studies using proxy indicators of frailty (outlined within Table 3).[17,83,84] Studies which use other measures and cite supporting evidence to suggest association with frailty will also be included. Accepting that these instruments have only moderate specificity for the identification of frailty and have yet to be validated within younger frail populations, they may be more appropriate to, and more commonly used in, younger groups.[17,85] Studies including participants described as frail with no quantitative measurement of this will be excluded.

For peer review only - http://bmjopen.bmj.com/site/about/guidelines.xhtml

Table 3. Functional measures which are recognised proxy measures of frailty, and published cut-offs indicative of frailty.[83,85,86,87,88]

Function test	Published cut points for the identification of frailty
Modified Physical Performance Test (PPT)	Score range: 0-36: • Not frail: 32-36 • Mild frailty: 25-32 • Moderate frailty: 17-24 • Dependent: <17
Balance Performance Oriented Mobility Assessment (BPOMA)	BPOMA>19
Short Physical Performance Battery (SPPB)	A score of ≤ 7 is indicative of frailty
Timed Get-Up-and-Go test (TUAG)	A score of ≥ 9 seconds
Gait speed test	A gait speed of 0.8m/s is indicative of frailty Taking more than 5 seconds to walk 4m
Sit to stand tests: • Sit to stand 10 seconds • Sit to stand 30 seconds • Sit to stand 60 seconds • Sit to stand 5 repetitions • Sit to stand 10 repetitions	Dependent upon the type of sit-stand test used: • ≥ 10 seconds for the five times sit stand • Lower than published criterion standards to maintain independence, stratified by age and gender for other forms of the test
Handgrip strength	Scores within the lowest quartile, stratified by sex
SARC- F questionnaire	Score of ≥ 4

Studies which target only carers will be included, providing they are delivering unpaid care for people with the above inclusion criteria and the interventions focus upon sedentary

For peer review only - http://bmjopen.bmj.com/site/about/guidelines.xhtml

behaviour, physical activity or exercise. Carer involvement is not included as a specific inclusion criterion, as we do not want to exclude relevant studies which did not include carers.

Concept

We define physical activity as “people moving, acting and performing within culturally specific spaces and contexts, and influenced by a unique array of interests, emotions, ideas, instructions and relationships”.[89] Within this, we include exercise interventions as ‘planned, structured, and repetitive bodily movement with the objective of improving or maintaining physical fitness’.[90] Multicomponent interventions, including rehabilitation programmes, will also be eligible, provided they meet the other inclusion criteria described. We will also include interventions which target sedentary behaviour, as previously defined.[18]

Context/settings

Studies from all settings will be eligible, including those in community, workplace, residential and hospital settings.

Searches will be limited to the year 2000 onwards to ensure they are relevant to current practice, and because the term ‘frailty’ as a medical condition of increased vulnerability is not well recognised prior to this.[91] Non-English language studies will be excluded.

Information sources

We will search for studies using the following databases and trials registries:

For systematic reviews: Cochrane; PROSPERO; Database of Abstracts of Reviews of Effects (DARE).

For published research: MEDLINE; EMBASE; Cumulative Index to Nursing and Allied Health (CINAHL); Web of Science; Sports Discus; PsycINFO; Pedro; Allied and Complementary Medicine (AMED); Cochrane Central Register of Controlled Trials (CENTRAL); Scopus.

For peer review only - http://bmjopen.bmj.com/site/about/guidelines.xhtml

For grey literature: internet searching (e.g., Google Scholar), BIOSIS previews, Open Grey and the Index to Scientific and Technical Proceedings.

Relevant ongoing clinical trials will be included if they provide sufficient information. The following databases will be searched for ongoing trials: CENTRAL; US National Institutes of Health Ongoing Trials Register (ClinicalTrials.gov); and the World Health Organization International Clinical Trials Registry Platform (ICTRP).

Meeting abstracts will not be included as they are unlikely to include the level of information required. Additional relevant literature will be identified via handsearching the references of included papers, drawing on forward and backwards citation tracking and electronic "cited by" searches using Google Scholar. Where included trials reference linked protocol papers or qualitative research, these will be included. Searches will be updated prior to publication.

Searches

Search terms used are outlined in Appendix 1. This strategy has been developed by a health information specialist and the review team for MEDLINE and will be translated for other databases.

Searches will be executed by HMLY with support from the information specialist where required. Initially MEDLINE (PubMed) and CINAHL will be searched to pilot the search strategy. Key words related to the aims of the review from the titles and abstract of retrieved papers, and of the index terms used to describe the articles will be reviewed. The research team will then meet to discuss any refinements before further searches are conducted.[79] An initial search for terms relating to frailty /function OR terms relating multimorbidity AND terms relating to physical activity/ sedentary behaviour yielded an unmanageable amount of data and, therefore the search was adjusted to frailty/ function terms AND multimorbidity terms AND physical activity/ sedentary behaviour terms.

For peer review only - http://bmjopen.bmj.com/site/about/guidelines.xhtml

3.Selection of evidence

Following de-duplication, the remaining studies will be independently screened for eligibility by two reviewers, using the inclusion criteria outlined above. A systematic approach will be facilitated by reference and review management software (EndNote V.X9 and Covidence).[79,81] Firstly, titles and abstracts will be screened to remove ineligible records. Following this, at least two reviewers will screen the remaining full-text copies. At this stage demographic and clinical characteristics of the studies will be assessed to determine if they meet the criteria for MLTCs. Any discrepancies will be resolved through discussion and the inclusion of an additional reviewer if required. Authors will be contacted if, after full-text screening, it is still unclear whether to include/exclude an article. The review team will meet regularly throughout each stage.

4.Data charting

A standardised data charting form will be used to comprehensively extract data from the included studies. The form will be developed by the review team based on the key objectives of the review. The form will be piloted by two reviewers on five studies to ensure all relevant information is extracted. Any changes to the form, and the rationale for these, will be recorded.

Microsoft Excel will be used to manage the extracted data. Where key information is missing from the full texts, authors will be contacted for additional information. The extraction of intervention characteristics, including how they are tailored and adapted will be guided by the template for intervention description and replication (TIDieR) checklist and the Consensus on Exercise Reporting Template (CERT).[92,93] For data relating to study methodology, relevant reporting guidelines will be used to guide the data extracted for each study type.[76] Key data to be extracted is outlined within Table 4. This list is not exhaustive, and data extracted may be subject to refinement.[76]

For peer review only - http://bmjopen.bmj.com/site/about/guidelines.xhtml

Table 4. Key data to be extracted within the review.

**Study details**	Author(s)
	Type of publication
	Year of publication
	Country of origin
**Description of methodology**	Aims/purpose
	Study design
	Inclusion and exclusion criteria
	Definition of frailty and frailty assessment or proxy functional measure used
	Primary and secondary outcomes
	Where applicable, definition of the carers involved
	Setting/ context (geographical, cultural, social environment and the organisational and political systems in which an intervention occurs)
	Sample size
	Characteristics of the study population, including:<br>Ethnicity<br>• Age<br>• Sex<br>• Indicators of socioeconomic status<br>• Presence of cognitive impairment<br>• Number, type and severity of long-term conditions<br>• Level of frailty of participants
	Where applicable, characteristics of carers or family members or significant others, including relationship to care receiver
**Description of**	Focus of the intervention (sedentary behaviour, physical activity, exercise or combination)
	If applicable, the type of physical activity/ exercise, including equipment used and an outline of the components included
	The methods used to prescribe of the intervention
	The decision rules for determining the starting level
	The intervention duration and dose i.e. the prescribed frequency and intensity, the duration of the intervention and any maintenance period

For peer review only

For peer review only - http://bmjopen.bmj.com/site/about/guidelines.xhtml

	The mode of delivery (face to face, virtual, individual or group)
	The decision rules for determining progression
	Details of how the program was progressed and how this was monitored
	The location/ setting of delivery (e.g., home-based or in-centre) including any necessary infrastructure or other relevant features
	Details, methods of, and reasons for tailoring, personalisation, or adaptation.
	Details and methods of any modifications to the intervention during the study, particularly in relation to periods of ill-health and fluctuating symptomology
	Intervention rationale, programme theory, or goals
	The physical or informational materials used in the intervention, including those provided to participants or used in intervention delivery or in training of intervention providers
	The procedures, activities, and/or processes used in the intervention, including any enabling or supportive activities, motivation strategies used (e.g. counselling/education; environmental modification; prompting; self-monitoring; social comparison; financial incentives)
	Amount of supervision, including contact time
	The intervention providers, including their qualifications/expertise, background and any training provided to them
	Description of how carers, relatives or significant others are included within the design, development, or delivery of the intervention
	How intervention fidelity was assessed, and by whom, including methods for measuring adherence will also be included
	Strategies used to maintain or improve fidelity
	How well the intervention was delivered as planned

For peer review only

For peer review only - http://bmjopen.bmj.com/site/about/guidelines.xhtml

To differentiate between physical activity and sedentary behaviour interventions, we will use the approach described by Hadgraft et al.[94] Finally, we will record the outcomes used to determine the interventions effectiveness, including outcomes relating to carer health and wellbeing, and the effects of the intervention on physical function and other relevant outcomes (particularly physical activity, sedentary behaviours and measures of quality of life). Key findings that relate to the scoping review questions will also be recorded alongside an assessment of the quality of the study.

Critical appraisal

Critical appraisal of the included studies will provide information on the quality of the available evidence. The identification of lower quality research will strengthen the identification of gaps within the existing literature.[79] Studies will not be excluded based on quality.

We will assess the quality of the included studies using the using the Mixed Methods Appraisal Tool (MMAT) which has previously been used in scoping reviews.[95,96,97]The MMAT is a brief, but reliable, critical appraisal tool which provides a single method for assessing the quality of a range of qualitative, quantitative and mixed-methods study designs.[76,98,99] Two reviewers will be familiarised with the tool and devise a strategy for applying the tool in a systematic manner.[98,99] Following this, they will undertake the assessment independently, with recourse to additional members of the team where required.[93] A detailed presentation of the ratings of each included criterion will be reported.[100]

5.Synthesis and reporting

We plan to use a convergent synthesis design, based upon each of the research questions identified.[101] Qualitative and quantitative data will be summarised using a narrative approach, supplemented by descriptive statistics, tables and figures as appropriate. Following this, qualitative and quantitative data relating to each question will be integrated using mixed-methods joint displays.[101] We anticipate that joint displays will be organised according to the

For peer review only - http://bmjopen.bmj.com/site/about/guidelines.xhtml

domains outlined within the TIDieR and CERT checklists.[92,93] Within the joint displays, consideration will also be given to the effectiveness of the identified interventions and the quality of the studies. This will enable us to better identify those interventions or components which appear to lead to more favourable outcomes, and to assess areas of ongoing uncertainty. The results of this review will be reported in accordance with PRISMA guidance.[82] This proposed synthesis plan will be further refined toward the end of the review.[76]

6.Stakeholder consultation

The final stage will involve discussing the review results with two stakeholder groups. The first will comprise approximately six to eight people living with both frailty and at least two co-existing long-term conditions from those included within this review, alongside with their carers/family members. These individuals are already engaged as members of a patient and public involvement (PPI) group for a study related to this review. We have already taken steps to ensure that we are actively considering equality diversity and inclusion and will continue to ensure the composition of this group is broadly representative of the population of interest.

The second group will comprise approximately six to eight exercise and healthcare professionals, and researchers with expertise in intervention development. These individuals are already engaged as collaborators for a study related to this review, or will be approached via existing links with local hospital and community health and research networks. We will include broad representation from professionals and academics with an interest in the management of MLTC and frailty alongside expertise in the speciality areas of interest in the review (respiratory, musculoskeletal, mental health, metabolic and infectious diseases, cardiology, neurology oncology and geriatrics).

The two groups will meet separately to mitigate any issues relating to power differentials, and to allow both groups to discuss their views openly. A single meeting for each group will be facilitated by HMLY. Meetings will not be audio-recorded but a co-facilitator will take notes

For peer review only - http://bmjopen.bmj.com/site/about/guidelines.xhtml

and observe group interactions. Meetings will be held face-to-face or virtually as circumstances allow. Given the heavy burden of healthcare and the increased potential for periods of illness, where lay stakeholders cannot attend, they will be consulted individually. Face to face meetings will be held in accessible locations. Materials will be adapted for the needs of those with sensory impairments as required. Lay members will be rewarded and recognised for their time and expertise in accordance with current guidance.[102]

The objective of the meetings will be to understand stakeholders' perspectives regarding any potential evidence informed interventions identified.[73] We will present a summary of our findings to inform discussions. They will be asked to consider how well any identified interventions fit the proposed context, and where and how they may need further adaptation.[74] Areas where there are ongoing uncertainties will be flagged. Stakeholders will also be asked for their views on areas which have not been addressed within the current evidence base which may be important to them. Following the meetings, a plan for future development and/or adaptation work will be developed and shared with the whole group for further comment. Feedback from the stakeholder consultation will be integrated with the findings of the review and described in the final report.

Patient and public involvement

In addition to shaping the research questions, PPI group members have provided feedback on how to make stakeholder meetings inclusive and accessible. They have also advised on the dissemination strategy adopted.

Ethics and dissemination

All data in this project will be gathered through database searches. Ethical approval to publish information from the stakeholder consultation process will be sought from the University of Leicester Internal Review Board for the stakeholder consultation stage of this review.

For peer review only - http://bmjopen.bmj.com/site/about/guidelines.xhtml

We will disseminate our findings at relevant academic and clinical meetings and by publishing them in an academic journal within the field. Results will also be disseminated to people living with both frailty and MLTC, and carers via the Lifestyle and Cardiovascular Biomedical Research Units involvement forum, relevant local and national patient charities, and our social media platforms.

Summary

The numbers of people living with MLTC are predicted to rise, and within this population those also living with frailty are particularly vulnerable to poor outcomes such as decreased function and independence. Sedentary behaviour, physical activity and exercise interventions form an integral part of chronic disease and frailty management and may also be important for those living with both MLTC and frailty. Existing systematic reviews suggest that exercise has the potential to improve function in those living with both MLTCs and frailty, but to date the focus has remained upon older people, which may not reflect those living with MLTC, who become frail at a younger age. Tailoring the intervention in response to symptoms and periods of ill-health and involving family members appears to be important in this population, but how this has been approached within existing studies is currently unknown. Additionally, the role of broader interventions which address sedentary behaviour are unclear. This scoping review aims to map the available evidence on the use of sedentary behaviour, physical activity and exercise interventions in people living with frailty and MLTCs. The results will inform the design of a tailored intervention and highlight gaps, directing future research.

Authors' contributions

HMLY conceived the study idea. HMLY, PD, LH,JS,FC,HS,PH,SL,MH, RP, SS,TY,MD designed the protocol and search strategy. HMLY, PD, LH,JS,FC,HS,PH,SL, MH,RP, SS,TY,MD prepared the manuscript. All authors reviewed final manuscript. Mentorship was provided by TY, SS, MD. Each author contributed important intellectual content during manuscript drafting or revision

For peer review only - http://bmjopen.bmj.com/site/about/guidelines.xhtml

and accepts accountability for the overall work ensuring that questions pertaining to the accuracy or integrity of any portion of the work are appropriately investigated and resolved.

Funding statement

The research was funded by the National Institute for Health Research (NIHR) Leicester Biomedical Research Centre, Collaboration for Leadership in Applied Health Research and Care East Midlands (CLAHRC EM). HMLY is supported by grants from the NIHR (NIHR301593). SJS. is supported by the Collaboration for Leadership in Applied Health Research and Care East Midlands. The views expressed in this publication are those of the authors and not necessarily those of the NHS, the NIHR or the Department of Health and Social Care. The funders had no role in the study design; collection, analysis, and interpretation of the data; writing the report; and the decision to submit the report for publication.

Competing interests' statement

All authors have no conflicts of interest to declare.

References

1. The Academy of Medical Sciences. *Multimorbidity: a priority for global health research* [online]. Available from https://acmedsci.ac.uk/file-download/82222577 [last accessed 20th November 2021].
2. Williams JS, Egede LE. The association between multimorbidity and quality of life, health status and functional disability. *The American Journal of the Medical Sciences*. 2016 Jul 1;352(1):45-52.
3. Jacob ME, Ni P, Driver J, Leritz E, Leveille SG, Jette AM, Bean JF. Burden and Patterns of Multi-morbidity: Impact on Disablement in Older Adults. *American Journal of Physical Medicine & Rehabilitation*. 2020 May;99(5):359.

For peer review only - http://bmjopen.bmj.com/site/about/guidelines.xhtml

4. Marengoni A, Angleman S, Fratiglioni L. Prevalence of disability according to multimorbidity and disease clustering: a population-based study. *Journal of Comorbidity.* 2011;1(1):11-8.

5. Nicholson K, Griffith LE, Sohel N, Raina P. Examining early and late onset of multimorbidity in the Canadian Longitudinal Study on Aging. *Journal of the American Geriatrics Society.* 2021 Jun;69(6):1579-91.

6. Quiñones AR, Markwardt S, Botoseneanu A. Multimorbidity combinations and disability in older adults. *Journals of Gerontology Series A: Biomedical Sciences and Medical Sciences.* 2016 Jun 1;71(6):823-30.

7. Read JR, Sharpe L, Modini M, Dear BF. Multimorbidity and depression: a systematic review and meta-analysis*. Journal of Affective Disorders*. 2017 Oct 15; 221:36-46.

8. Stafford M, Steventon A, Thorlby R, Fisher R, Turton C, Deeny S. Briefing: Understanding the health care needs of people with multiple health conditions [online]. Health Foundation; 2017.Available from: file:///C:/Users/Hannah/Downloads/Understanding%20the%20health%20care%20needs%20of%20people%20with%20multiple%20health%20conditions%20(1).pdf [Last accessed 10th December 2021].

9. Sibley KM, Voth J, Munce SE, Straus SE, Jaglal SB. Chronic disease and falls in community-dwelling Canadians over 65 years old: a population-based study exploring associations with number and pattern of chronic conditions. *BMC Geriatrics*. 2014 Dec;14(1):1-1.

10. Clegg A, Young J, Iliffe S, Rikkert MO, Rockwood K. Frailty in elderly people. *The Lancet.* 2013 Mar 2;381(9868):752-62.

11. Aarts S, Patel KV, Garcia ME, Van den Akker M, Verhey FR, Metsemakers JF, Van Boxtel MP, Gudnason V, Jonsdottir MK, Siggeirsdottir K, Jonsson PV. Co-presence of multimorbidity and disability with frailty: an examination of heterogeneity in the frail older population. *The Journal of Frailty & Aging*. 2015;4(3):131.

12. Villacampa-Fernandez P, Navarro-Pardo E, Tarin JJ, Cano A. Frailty and multimorbidity: Two related yet different concepts. *Maturitas.* 2017 Jan 1; 95:31-5.

13. Espinoza SE, Quiben M, Hazuda HP. Distinguishing comorbidity, disability, and frailty. *Current Geriatrics Reports.* 2018 Dec;7(4):201-9.

For peer review only - http://bmjopen.bmj.com/site/about/guidelines.xhtml

14. Aprahamian I, Mamoni RL, Cervigne NK, Augusto TM, Romanini CV, Petrella M, da Costa DL, Lima NA, Borges MK, Voshaar RC. Design and protocol of the multimorbidity and mental health cohort study in frailty and aging (MiMiCS-FRAIL): unraveling the clinical and molecular associations between frailty, somatic disease burden and late life depression. *BMC Psychiatry*. 2020 Dec;20(1):1-1.
15. Hanlon P, Nicholl BI, Jani BD, Lee D, McQueenie R, Mair FS. Frailty and pre-frailty in middle-aged and older adults and its association with multimorbidity and mortality: a prospective analysis of 493 737 UK Biobank participants. *The Lancet Public Health*. 2018 Jul 1;3(7): e323-32.
16. Whitty C J M, MacEwen C, Goddard A, Alderson D, Marshall M, Calderwood C et al. Rising to the challenge of multimorbidity *BMJ* 2020; 368 :l6964 doi:10.1136/bmj.l6964
17. Spiers GF, Kunonga TP, Hall A, Beyer F, Boulton E, Parker S, Bower P, Craig D, Todd C, Hanratty B. Measuring frailty in younger populations: a rapid review of evidence. *BMJ Open.* 2021 Mar 1;11(3):e047051.
18. Tremblay MS, Aubert S, Barnes JD, Saunders TJ, Carson V, Latimer-Cheung AE, Chastin SF, Altenburg TM, Chinapaw MJ. Sedentary behavior research network (SBRN)–terminology consensus project process and outcome. *International Journal of Behavioral Nutrition and Physical Activity*. 2017 Dec;14(1):1-7.
19. Dempsey PC, Biddle SJ, Buman MP, Chastin S, Ekelund U, Friedenreich CM, Katzmarzyk PT, Leitzmann MF, Stamatakis E, van der Ploeg HP, Willumsen J. New global guidelines on sedentary behaviour and health for adults: broadening the behavioural targets. *International Journal of Behavioral Nutrition and Physical Activity*. 2020 Dec;17(1):1-2.
20. Lee WC, Ory MG. The engagement in physical activity for middle-aged and older adults with multiple chronic conditions: findings from a community health assessment. *Journal of Aging Research.* 2013 Sep 5;2013.
21. Dempsey PC, Friedenreich CM, Leitzmann MF, Buman MP, Lambert E, Willumsen J, Bull F. Global Public Health Guidelines on Physical Activity and Sedentary Behavior for People Living With Chronic Conditions: A Call to Action. *Journal of Physical Activity and Health.* 2020 Dec 4;18(1):76-85.
22. Vancampfort D, Koyanagi A, Ward PB, Rosenbaum S, Schuch FB, Mugisha J, Richards J, Firth J, Stubbs B. Chronic physical conditions, multimorbidity and physical activity

For peer review only - http://bmjopen.bmj.com/site/about/guidelines.xhtml

across 46 low-and middle-income countries. *International Journal of Behavioral Nutrition and Physical Activity.* 2017 Dec;14(1):1-3.

23. Dodds RM, Granic A, Robinson SM, Sayer AA. Sarcopenia, long-term conditions, and multimorbidity: findings from UK Biobank participants. *Journal of cachexia, sarcopenia and muscle.* 2020 Feb;11(1):62-8.
24. Hurst C, Murray JC, Granic A, Hillman SJ, Cooper R, Sayer AA, Robinson SM, Dodds RM. Long-term conditions, multimorbidity, lifestyle factors and change in grip strength over 9 years of follow-up: Findings from 44,315 UK biobank participants. *Age and ageing.* 2021 Nov;50(6):2222-9.
25. Cassidy S, Fuller H, Chau J, Catt M, Bauman A, Trenell MI. Accelerometer-derived physical activity in those with cardio-metabolic disease compared to healthy adults: a UK Biobank study of 52,556 participants. *Acta Diabetologica*. 2018 Sep;55(9):975-9.
26. Steeves JA, Shiroma EJ, Conger SA, Van Domelen D, Harris TB. Physical activity patterns and multimorbidity burden of older adults with different levels of functional status: NHANES 2003–2006. *Disability and Health Journal*. 2019 Jul 1;12(3):495-502.
27. Autenrieth CS, Kirchberger I, Heier M, Zimmermann AK, Peters A, Döring A, Thorand B. Physical activity is inversely associated with multimorbidity in elderly men: results from the KORA-Age Augsburg Study. *Preventive Medicine*. 2013 Jul 1;57(1):17-9.
28. Martinez-Gomez D, Guallar-Castillon P, Garcia-Esquinas E, Bandinelli S, Rodríguez-Artalejo F. Physical activity, and the effect of multimorbidity on all-cause mortality in older adults. *Mayo Clinic Proceedings* 2017 Mar 1 (Vol. 92, No. 3, pp. 376-382).
29. Chudasama YV, Khunti KK, Zaccardi F, Rowlands AV, Yates T, Gillies CL, Davies MJ, Dhalwani NN. Physical activity, multimorbidity, and life expectancy: a UK Biobank longitudinal study. *BMC Medicine*. 2019 Dec 1;17(1):108.
30. Cruz-Jentoft AJ, Sayer AA. Sarcopenia. The Lancet. 2019 Jun 29;393(10191):2636-46.
31. Fried LP, Tangen CM, Walston J, Newman AB, Hirsch C, Gottdiener J, Seeman T, Tracy R, Kop WJ, Burke G, McBurnie MA. Frailty in older adults: evidence for a phenotype. *The Journals of Gerontology Series A: Biological Sciences and Medical Sciences*. 2001 Mar 1;56(3):M146-57.
32. World Health Organisation. *Global action plan on physical activity 2018–2030: more active people for a healthier world.* Geneva: World Health Organization; Report number: CC BY-NC-SA 3.0 IGO, 2018.

For peer review only - http://bmjopen.bmj.com/site/about/guidelines.xhtml

33. Rogers NT, Marshall A, Roberts CH, Demakakos P, Steptoe A, Scholes S. Physical activity, and trajectories of frailty among older adults: Evidence from the English Longitudinal Study of Ageing. *PloS One*. 2017 Feb 2;12(2): e0170878.
34. Peterson MJ, Giuliani C, Morey MC, Pieper CF, Evenson KR, Mercer V, Cohen HJ, Visser M, Brach JS, Kritchevsky SB, Goodpaster BH. Physical activity as a preventative factor for frailty: the health, aging, and body composition study. *The Journals of Gerontology: Series A.* 2009 Jan 1;64(1):61-8.
35. da Silva Coqueiro R, de Queiroz BM, Oliveira DS, das Merces MC, JA OC, Pereira R, Fernandes MH. Cross-sectional relationships between sedentary behavior and frailty in older adults. *The Journal of Sports Medicine and Physical Fitness*. 2016 Mar 9;57(6):825-30.
36. Yuan L, Chang M, Wang J. Abdominal obesity, body mass index and the risk of frailty in community-dwelling older adults: a systematic review and meta-analysis. *Age and Ageing.* 2021 Mar 10.
37. Lu Y, Liu S, Qiao Y, Li G, Wu Y, Ke C. Waist-to-height ratio, waist circumference, body mass index, waist divided by height0. 5 and the risk of cardiometabolic multimorbidity: a national longitudinal cohort study. *Nutrition, Metabolism and Cardiovascular Diseases.* 2021 Jun 4.
38. Pollack LM, Wang M, Leung MY, Colditz G, Herrick C, Chang SH. Obesity-related multimorbidity and risk of cardiovascular disease in the middle-aged population in the United States. *Preventive Medicine*. 2020 Oct 1;139:106225.
39. Ford JC, Ford JA. Multimorbidity: will it stand the test of time? *Age and Ageing*. 2018 Jan 1;47(1):6-8.
40. National Institute for Health and Care Excellence. *Dementia, disability, and frailty in later life – mid-life approaches to delay or prevent onset*. 2015 [online]. Available from: https://www.nice.org.uk/guidance/ng16 [last accessed 10th December 2021]
41. National Institute for Health and Care Excellence. *Multimorbidity:clinical assessment and management.* NICE guideline 2016 [online]. Available from https://www.nice.org.uk/guidance/ng56 [last accessed 10th December 2021]
42. Kingston A, Robinson L, Booth H, Knapp M, Jagger C, MODEM project. Projections of multi-morbidity in the older population in England to 2035: estimates from the

For peer review only - http://bmjopen.bmj.com/site/about/guidelines.xhtml

Population Ageing and Care Simulation (PACSim) model. *Age and ageing*. 2018 May 1;47(3):374-80.

43. Frost R, Rait G, Wheatley A, Wilcock J, Robinson L, Harrison Dening K, Allan L, Banerjee S, Manthorpe J, Walters K, PriDem Study project team. What works in managing complex conditions in older people in primary and community care? A state-of-the-art review. *Health & Social Care in the Community*. 2020 Nov;28(6):1915-27.
44. Public Health England. *Wider impacts of COVID-19 on physical activity, deconditioning and falls in older adults* [online]. 2021 Available from: https://assets.publishing.service.gov.uk/government/uploads/system/uploads/attachment_data/file/1010501/HEMT_Wider_Impacts_Falls.pdf [Last accessed 29th October 2021].
45. De Vries NM, Van Ravensberg CD, Hobbelen JS, Rikkert MO, Staal JB, Nijhuis-Van der Sanden MW. Effects of physical exercise therapy on mobility, physical functioning, physical activity and quality of life in community-dwelling older adults with impaired mobility, physical disability and/or multi-morbidity: a meta-analysis. *Ageing Research Reviews.* 2012 Jan 1;11(1):136-49.
46. Bricca A, Harris LK, Saracutu M, Smith SM, Juhl CB, Skou ST. Benefits and harms of exercise therapy in people with multimorbidity: a systematic review and meta-analysis of randomised controlled trials. *Ageing Research Reviews*. 2020 Sep 5:101166.
47. Crowe M, Jordan J, Burrell B, Jones V, Gillon D, Harris S, Wilkinson A. Clinical effectiveness of transdiagnostic health management interventions for older people with multimorbidity: a quantitative systematic review. *Journal of Advanced Nursing.* 2016 Oct;72(10):2315-29.
48. Talar K, Hernández-Belmonte A, Vetrovsky T, Steffl M, Kałamacka E, Courel-Ibáñez J. Benefits of Resistance Training in Early and Late Stages of Frailty and Sarcopenia: A Systematic Review and Meta-Analysis of Randomized Controlled Studies. *Journal of Clinical Medicine*. 2021 Jan;10(8):1630.
49. Campbell E, Petermann-Rocha F, Welsh P, Celis-Morales C, Pell JP, Ho FK, Gray SR. The effect of exercise on quality of life and activities of daily life in frail older adults: A systematic review of randomised control trials. *Experimental Gerontology*. 2021 Feb 18:111287.

For peer review only - http://bmjopen.bmj.com/site/about/guidelines.xhtml

50. Tuttle LJ, Bittel DC, Bittel AJ, Sinacore DR. Early-onset physical frailty in adults with diabesity and peripheral neuropathy. *Canadian Journal of Diabetes*. 2018 Oct 1;42(5):478-83.

51. Gebregziabher M, Ward RC, Taber DJ, Walker RJ, Ozieh M, Dismuke CE, Axon RN, Egede LE. Ethnic and geographic variations in multimorbidty: Evidence from three large cohorts. *Social Science & Medicine*. 2018 Aug 1;211:198-206.

52. Parker SG, Corner L, Laing K, Nestor G, Craig D, Collerton J, Frith J, Roberts HC, Sayer AA, Allan LM, Robinson L. Priorities for research in multiple conditions in later life (multi-morbidity): findings from a James Lind alliance priority setting partnership. *Age and Ageing.* 2019 May 1;48(3):401-6.

53. Smith SM, Wallace E, O'Dowd T, Fortin M. Interventions for improving outcomes in patients with multimorbidity in primary care and community settings. *Cochrane Database of Systematic Reviews* 2021, Issue 1. Art. No.: CD006560. DOI: 10.1002/14651858.CD006560.pub4. Accessed 22 October 2021.

54. Reid H, Ridout AJ, Tomaz SA, Kelly P, Jones N. Benefits outweigh the risks: a consensus statement on the risks of physical activity for people living with long-term conditions. *British Journal of Sports Medicine.* 2021 Oct 14.

55. Maddocks M, Kon SS, Canavan JL, Jones SE, Nolan CM, Labey A, Polkey MI, Man WD. Physical frailty and pulmonary rehabilitation in COPD: a prospective cohort study. *Thorax.* 2016 Nov 1;71(11):988-95.

56. Young HM, March DS, Highton PJ, Graham-Brown MP, Churchward DC, Grantham C, Goodliffe S, Jones W, Cheung MM, Greenwood SA, Eborall HC. Exercise for people living with frailty and receiving haemodialysis: a mixed-methods randomised controlled feasibility study. *BMJ Open.* 2020 Nov 1;10(11):e041227.

57. Blackstock FC, ZuWallack R, Nici L, Lareau SC. Why don't our patients with chronic obstructive pulmonary disease listen to us? The enigma of nonadherence. *Annals of the American Thoracic Society.* 2016 Mar;13(3):317-23.

58. Mañas A, del Pozo-Cruz B, García-García FJ, Guadalupe-Grau A, Ara I. Role of objectively measured sedentary behaviour in physical performance, frailty and mortality among older adults: A short systematic review. *European Journal of Sport Science.* 2017 Aug 9;17(7):940-53.

For peer review only - http://bmjopen.bmj.com/site/about/guidelines.xhtml

59. Prince SA, Saunders TJ, Gresty K, Reid RD. A comparison of the effectiveness of physical activity and sedentary behaviour interventions in reducing sedentary time in adults: a systematic review and meta-analysis of controlled trials. *Obesity Reviews.* 2014 Nov;15(11):905-19.

60. Kehler DS, Theou O. The impact of physical activity and sedentary behaviors on frailty levels. *Mechanisms of Ageing and Development.* 2019 Jun 1;180:29-41.

61. Dogra S, Copeland JL, Altenburg TM, Heyland DK, Owen N, Dunstan DW. Start with reducing sedentary behavior: A stepwise approach to physical activity counseling in clinical practice. *Patient Education and Counseling*. 2021 Sep 13.

62. Lerma NL, Cho CC, Swartz AM, Miller NE, Keenan KG, Strath SJ. Isotemporal substitution of sedentary behavior and physical activity on function. *Medicine and Science in Sports and Exercise*. 2018 Apr;50(4):792.

63. Young HML, Goodliffe S, Helen C E, Simon C, Greenwood S, Singh SJ, Smith A, Burton J. SP418 Frailty and falls: A qualitative study exploring the experiences of patients living with end-stage renal disease and haemodialysis. *Nephrology Dialysis Transplantation.* 2019 Jun 1;34(Supplement_1):gfz103-SP418.

64. Ellis KR, Hecht HK, Young TL, Oh S, Thomas S, Hoggard LS, Ali Z, Olawale R, Carthron D, Corbie-Smith G, Eng E. Peer Reviewed: Chronic Disease Among African American Families: A Systematic Scoping Review. *Preventing Chronic Disease.* 2020;17.

65. Singh K, Patel SA, Biswas S, Shivashankar R, Kondal D, Ajay VS, Anjana RM, Fatmi Z, Ali MK, Kadir MM, Mohan V. Multimorbidity in South Asian adults: prevalence, risk factors and mortality. *Journal of Public Health*. 2019 Mar 1;41(1):80-9.

66. Polenick CA, Birditt KS, Turkelson A, Kales HC. Chronic condition discordance and physical activity among midlife and older couples. *Health Psychology*. 2021 Jan;40(1):11.

67. Polenick CA, Birditt KS, Turkelson A, Kales HC. Individual-level and couple-level discordant chronic conditions: Longitudinal links to functional disability. *Annals of Behavioral Medicine*. 2020 Jul;54(7):455-69.

68. Gomes GA, Luchesi BM, Gratão AC, Orlandi FD, Say KG, Inouye K, Alexandre TD, Brigola AG, Pavarini SC. Prevalence of physical inactivity and associated factors among older caregivers of older adults. *Journal of Aging and Health.* 2019 Jun;31(5):793-813.

For peer review only - http://bmjopen.bmj.com/site/about/guidelines.xhtml

69. Greaney ML, Kunicki ZJ, Drohan MM, Ward-Ritacco CL, Riebe D, Cohen SA. Self-reported changes in physical activity, sedentary behavior, and screen time among informal caregivers during the COVID-19 pandemic. *BMC Public Health*. 2021 Dec;21(1):1-9.
70. Doyle KL, Toepfer M, Bradfield AF, Noffke A, Ausderau KK, Andreae S, Pickett KA. Systematic review of exercise for caregiver–care recipient dyads: What is best for spousal caregivers—exercising together or not at all? *The Gerontologist*. 2021 Sep;61(6):e283-301.
71. Munn Z, Peters MD, Stern C, Tufanaru C, McArthur A, Aromataris E. Systematic review or scoping review? Guidance for authors when choosing between a systematic or scoping review approach. *BMC Medical Research Methodology.* 2018 Dec;18(1):1-7.
72. O'Cathain A, Croot L, Duncan E, Rousseau N, Sworn K, Turner KM, Yardley L, Hoddinott P. Guidance on how to develop complex interventions to improve health and healthcare. *BMJ Open.* 2019 Aug 1;9(8):e029954.
73. O’Cathain A, Croot L, Sworn K, Duncan E, Rousseau N, Turner K, Yardley L, Hoddinott P. Taxonomy of approaches to developing interventions to improve health: a systematic methods overview. *Pilot and Feasibility Studies*. 2019a Dec;5(1):1-27.
74. Moore G, Campbell M, Copeland L, Craig P, Movsisyan A, Hoddinott P, Littlecott H, O’Cathain A, Pfadenhauer L, Rehfuess E, Segrott J. Adapting interventions to new contexts—the ADAPT guidance. *BMJ.* 2021 Aug 3;374.
75. Skivington K, Matthews L, Simpson SA, Craig P, Baird J, Blazeby JM, Boyd KA, Craig N, French DP, McIntosh E, Petticrew M. A new framework for developing and evaluating complex interventions: update of Medical Research Council guidance. *BMJ*. 2021 Sep 30;374.
76. Noyes J, Booth A, Moore G, Flemming K, Tunçalp Ö, Shakibazadeh E. Synthesising quantitative and qualitative evidence to inform guidelines on complex interventions: clarifying the purposes, designs and outlining some methods. *BMJ Global Health*. 2019 Jan 1;4(Suppl 1):e000893.
77. Petticrew M, Rehfuess E, Noyes J, Higgins JP, Mayhew A, Pantoja T, Shemilt I, Sowden A. Synthesizing evidence on complex interventions: how meta-analytical, qualitative, and mixed-method approaches can contribute. *Journal of Clinical Epidemiology*. 2013 Nov 1;66(11):1230-43.

For peer review only - http://bmjopen.bmj.com/site/about/guidelines.xhtml

78. Arksey H, O'Malley L. Scoping studies: towards a methodological framework. *International Journal of Social Research Methodology*. 2005 Feb 1;8(1):19-32.
79. Levac D, Colquhoun H, O'Brien KK. Scoping studies: advancing the methodology. *Implementation Science.* 2010 Dec;5(1):1-9.
80. Colquhoun HL, Levac D, O'Brien KK, Straus S, Tricco AC, Perrier L, Kastner M, Moher D. Scoping reviews: time for clarity in definition, methods, and reporting. *Journal of Clinical Epidemiology.* 2014 Dec 1;67(12):1291-4.
81. Daudt HM, van Mossel C, Scott SJ. Enhancing the scoping study methodology: a large, inter-professional team's experience with Arksey and O'Malley's framework. *BMC Medical Research Methodology.* 2013 Dec;13(1):1-9.
82. Tricco AC, Lillie E, Zarin W, O'Brien KK, Colquhoun H, Levac D, Moher D, Peters MD, Horsley T, Weeks L, Hempel S. PRISMA extension for scoping reviews (PRISMA-ScR): checklist and explanation. *Annals of Internal Medicine*. 2018 Oct 2;169(7):467-73.
83. Bouillon K, Kivimaki M, Hamer M, Sabia S, Fransson EI, Singh-Manoux A, Gale CR, Batty GD. Measures of frailty in population-based studies: an overview. *BMC Geriatrics.* 2013 Dec;13(1):1-1.
84. De Vries NM, Staal JB, Van Ravensberg CD, Hobbelen JS, Rikkert MO, Nijhuis-Van der Sanden MW. Outcome instruments to measure frailty: a systematic review. *Ageing Research Reviews.* 2011 Jan 1;10(1):104-14.
85. British Geriatric Society. *Fit for frailty* [online]. 2014 Available from: https://www.bgs.org.uk/resources/recognising-frailty [Last accessed 17th November 2021].
86. Studenski S, Perera S, Patel K, Rosano C, Faulkner K, Inzitari M, Brach J, Chandler J, Cawthon P, Connor EB, Nevitt M. Gait speed and survival in older adults. *JAMA*. 2011 Jan 5;305(1):50-8.
87. Makizako H, Shimada H, Doi T, Tsutsumimoto K, Nakakubo S, Hotta R, Suzuki T. Predictive cutoff values of the five-times sit-to-stand test and the timed "up & go" test for disability incidence in older people dwelling in the community. *Physical Therapy*. 2017 Apr 1;97(4):417-24.
88. Bahat G, Ozkok S, Kilic C, Karan MA. SARC-F questionnaire detects frailty in older adults. *The Journal of Nutrition, Health & Aging*. 2021 Apr;25(4):448-53.

For peer review only - http://bmjopen.bmj.com/site/about/guidelines.xhtml

89. Piggin J. What is physical activity? A holistic definition for teachers, researchers and policy makers. *Frontiers in Sports and Active Living.* 2020 Jun 18;2:72.

90. Caspersen CJ, Powell KE, Christenson GM. Physical activity, exercise, and physical fitness: definitions and distinctions for health-related research. *Public Health Reports.* 1985 Mar;100(2):126.

91. Rockwood, K., Howlett, S.E. Fifteen years of progress in understanding frailty and health in aging. *BMC Med* **16,** 220 (2018). Available from: https://doi.org/10.1186/s12916-018-1223-3

92. Hoffmann TC, Glasziou PP, Boutron I, Milne R, Perera R, Moher D, Altman DG, Barbour V, Macdonald H, Johnston M, Lamb SE. Better reporting of interventions: template for intervention description and replication (TIDieR) checklist and guide. *BMJ*. 2014 Mar 7;348.

93. Slade SC, Dionne CE, Underwood M, Buchbinder R. Consensus on exercise reporting template (CERT): explanation and elaboration statement. *British Journal of Sports Medicine.* 2016 Dec 1;50(23):1428-37.

94. Hadgraft NT, Winkler E, Climie RE, Grace MS, Romero L, Owen N, Dunstan D, Healy G, Dempsey PC. Effects of sedentary behaviour interventions on biomarkers of cardiometabolic risk in adults: systematic review with meta-analyses*. British journal of sports medicine.* 2021 Feb 1;55(3):144-54.

95. Puts MT, Toubasi S, Andrew MK, Ashe MC, Ploeg J, Atkinson E, Ayala AP, Roy A, Rodríguez Monforte M, Bergman H, McGilton K. Interventions to prevent or reduce the level of frailty in community-dwelling older adults: a scoping review of the literature and international policies. *Age and Ageing.* 2017 May 1;46(3):383-92.

96. Pace R, Pluye P, Bartlett G, Macaulay AC, Salsberg J, Jagosh J, Seller R. Testing the reliability and efficiency of the pilot Mixed Methods Appraisal Tool (MMAT) for systematic mixed studies review. *International Journal of Nursing Studies*. 2012 Jan 1;49(1):47-53.

97. Pluye P, Gagnon MP, Griffiths F, Johnson-Lafleur J. A scoring system for appraising mixed methods research, and concomitantly appraising qualitative, quantitative and mixed methods primary studies in mixed studies reviews. *International Journal of Nursing Studies*. 2009 Apr 1;46(4):529-46.

For peer review only - http://bmjopen.bmj.com/site/about/guidelines.xhtml

98. Souto RQ, Khanassov V, Hong QN, Bush PL, Vedel I, Pluye P. Systematic mixed studies reviews: updating results on the reliability and efficiency of the mixed methods appraisal tool. *International Journal of Nursing Studies*. 2015 Jan 1;52(1):500-1.

99. Hong QN, Gonzalez-Reyes A, Pluye P. Improving the usefulness of a tool for appraising the quality of qualitative, quantitative and mixed methods studies, the Mixed Methods Appraisal Tool (MMAT). *Journal of Evaluation in Clinical Practice*. 2018 Jun;24(3):459-67.

100. Hong QN, Pluye P, Fàbregues S, Bartlett G, Boardman F, Cargo M, Dagenais P, Gagnon M-P, Griffiths F, Nicolau B, O'Cathain A, Rousseau M-C, Vedel I. *Mixed Methods Appraisal Tool (MMAT)*. Canadian Intellectual Property Office, Industry Canada. Report number: 1148552, 2018.

101. Creswell JW and Plano Clark VL. Designing and Conducting Mixed Methods Research. 3rd Ed. United States: Sage International; 2017.

102. National Institute for Health Research. *Payment guidance for researchers and professionals* [online]. 2020 Available from https://www.nihr.ac.uk/documents/payment-guidance-for-researchers-and-professionals/27392 [last accessed 23rd October 2021].

For peer review only - http://bmjopen.bmj.com/site/about/guidelines.xhtml

For peer review only

For peer review only - http://bmjopen.bmj.com/site/about/guidelines.xhtml

Appendix 1. MEDLINE Search Strategy

1	exp comorbidity/
2	comorbid$.mp.
3	co-morbid$.mp.
4	exp multimorbidity/
5	multimorbid$.mp.
6	multi-morbid$.mp.
7	multidisease?.mp.
8	multi-disease?.mp.
9	exp Multiple Chronic Conditions/
10	Multiple Chronic Conditions.mp.
11	poly-morbidit*.mp.
12	polymorbidit*.mp.
13	poly-patholog*.mp.
14	polypatholog*.mp.
15	pluripatholog*.mp.
16	pluri-patholog*.mp.
17	multipatholog*.mp.
18	multi-patholog*.mp.
19	multicondition.mp.

For peer review only

20	multi-condition.mp.
21	exp Syndemic/
22	syndemic.mp.
23	"multiple health".mp.
24	"multiple chronic".mp.
25	"multiple long-term".mp.
26	condition*.mp.
27	ill*.mp.
28	disorder*.mp.
29	disease*.mp.
30	23 or 24 or 25
31	26 or 27 or 28 or 29
32	30 and 31
33	exp Diabetes Mellitus, Type 2/
34	diabetes type 2.mp.
35	diabetes type II.mp.
36	early onset diabetes.mp.
37	(diabetes adj3 type 2).mp. [mp=title, abstract, original title, name of substance word, subject heading word, floating sub-heading word, keyword heading word, organism supplementary concept word,

For peer review only

	protocol supplementary concept word, rare disease supplementary concept word, unique identifier, synonyms]
38	(diabetes adj2 type ii).mp. [mp=title, abstract, original title, name of substance word, subject heading word, floating sub-heading word, keyword heading word, organism supplementary concept word, protocol supplementary concept word, rare disease supplementary concept word, unique identifier, synonyms]
39	(diabetes adj type two).mp. [mp=title, abstract, original title, name of substance word, subject heading word, floating sub-heading word, keyword heading word, organism supplementary concept word, protocol supplementary concept word, rare disease supplementary concept word, unique identifier, synonyms]
40	T2DM.mp.
41	T2D.mp.
42	33 or 34 or 35 or 36 or 37 or 38 or 39 or 40 or 41
43	exp Hypertension/
44	exp Blood Pressure/
45	Blood Pressure.mp.
46	hypertens*.mp.
47	43 or 44 or 45 or 46
48	exp Heart Diseases/
49	exp Heart Failure/
50	exp Cardiovascular Diseases/

For peer review only

51	exp Coronary Disease/
52	cardiac.mp.
53	heart.mp.
54	cardiovascular.mp.
55	coronary.mp.
56	52 or 53 or 54 or 55
57	31 and 56
58	48 or 49 or 50 or 51 or 57
59	exp Vascular Diseases/
60	exp Carotid Artery Diseases/
61	cerebrovascular.mp.
62	vascular.mp.
63	carotoid$.mp.
64	arter$.mp.
65	61 or 62 or 63 or 64
66	31 and 65
67	exp Cerebrovascular Disorders/
68	59 or 60 or 66 or 67
69	exp Asthma/
70	asthma*.mp.

For peer review only

71	69 or 70
72	exp Pulmonary Disease, Chronic Obstructive/
73	COPD.mp.
74	chronic obstructive pulmonary disease.mp.
75	72 or 73 or 74
76	exp Hyperlipidemias/
77	exp Hypercholesterolemia/
78	exp Hypertriglyceridemia/
79	hyperlipid*em*.mp.
80	hypercholesterol*emia$.mp.
81	hypertriglycerid*emia*.mp.
82	76 or 77 or 78 or 79 or 80 or 81
83	exp Arthritis/
84	exp Arthritis, Rheumatoid/
85	exp Osteoarthritis/
86	arthritis.mp.
87	rheumatoid arthritis.mp.
88	osteoarthritis.mp.
89	83 or 84 or 85 or 86 or 87 or 88
90	exp Depression/

For peer review only

91	exp Anxiety/
92	depression.mp.
93	anxiety.mp.
94	90 or 91 or 92 or 93
95	exp Neoplasms/
96	malignan*.mp.
97	cancer.mp.
98	95 or 96 or 97
99	exp HIV/
100	exp Acquired Immunodeficiency Syndrome/
101	Acquired Immunodeficiency Syndrome.mp.
102	AIDS.mp.
103	HIV.mp.
104	99 or 100 or 101 or 102 or 103
105	exp Kidney Failure, Chronic/
106	exp Kidney Diseases/
107	exp Renal Insufficiency/
108	renal.mp.
109	kidney.mp.
110	108 or 109

For peer review only

111	31 and 110
112	105 or 106 or 107 or 111
113	exp Liver Diseases/
114	liver.mp.
115	31 and 114
116	113 or 115
117	exp Osteoporosis/
118	osteoporosis.mp.
119	117 or 118
120	exp Obesity/
121	obes*.mp.
122	120 or 121
123	exp Multiple Sclerosis/
124	multiple sclerosis.mp.
125	"MS".mp.
126	123 or 124 or 125
127	exp Parkinson Disease/
128	parkinsons disease.mp.
129	"PD".mp.
130	127 or 128 or 129

For peer review only

131	peripheral artery disease.mp.
132	exp Peripheral Arterial Disease/
133	131 or 132
134	co-occur*.mp.
135	cooccur*.mp.
136	concurrent.mp.
137	coexist*.mp.
138	co-exist*.mp.
139	134 or 135 or 136 or 137 or 138
140	31 and 139
141	1 or 2 or 3 or 4 or 5 or 6 or 7 or 8 or 9 or 10 or 11 or 12 or 13 or 14 or 15 or 16 or 17 or 18 or 19 or 20 or 21 or 22 or 32
142	42 or 47 or 58 or 68 or 71 or 75 or 82 or 89 or 94 or 98 or 104 or 112 or 116 or 119 or 122 or 126 or 130 or 133
143	140 and 142
144	141 or 143
145	exp Frail Elderly/
146	exp Frailty/
147	frail*.mp.
148	prefrail.mp.

For peer review only

149	pre-frail.mp.
150	functionally-impaired.mp.
151	Physical function*.mp.
152	"Physical frail*".mp.
153	Debility.mp.
154	145 or 146 or 147 or 148 or 149 or 150 or 151 or 152 or 153
155	exp Exercise/
156	exp Physical Fitness/
157	exp Sedentary Behavior/
158	exp Rehabilitation/
159	exp Exercise Therapy/
160	exercis*.mp.
161	physical activit*.mp.
162	physical fitness.mp.
163	sedentary behavio*r.mp.
164	sedentary.mp.
165	rehabilitat*.mp.
166	exercise therapy.mp.
167	155 or 156 or 157 or 158 or 159 or 160 or 161 or 162 or 163 or 164 or 165 or 166

For peer review only

168	144 and 154 and 167
169	limit 168 to yr="2000 -Current"

For peer review only

BMJ Open

Physical activity and sedentary behaviour interventions for people living with both frailty and multiple long-term conditions: a scoping review protocol

Journal:	*BMJ Open*
Manuscript ID	bmjopen-2022-061104.R1
Article Type:	Protocol
Date Submitted by the Author:	11-Mar-2022
Complete List of Authors:	Young, Hannah; University Hospitals of Leicester NHS Trust, Leicester Diabetes Centre, College of Life Sciences; University of Leicester, Diabetes Research Centre<br>Yates, Thomas; University of Leicester, Diabetes Research Centre, College of Life Sciences<br>Dempsey, Paddy ; University of Leicester, Diabetes Research Centre, College of Life Sciences; Cambridge Biomedical Campus, MRC Epidemiology Unit, Institute of Metabolic Science<br>Herring, Louisa ; University Hospitals of Leicester NHS Trust, Leicester Diabetes Centre,<br>Henson, Joseph; University Hospitals of Leicester NHS Trust, Leicester Diabetes Centre; University of Leicester, Diabetes Research Centre, College of Life Sciences,<br>Sargeant, Jack; University Hospitals of Leicester NHS Trust, Leicester Diabetes Centre; University of Leicester, Diabetes Research Centre, College of Life Sciences,<br>Curtis, Ffion; University Hospitals of Leicester NHS Trust, NIHR Applied Research Collaboration East Midlands<br>Sathanapally, Harini; University Hospitals of Leicester NHS Trust, NIHR Applied Research Collaboration East Midlands<br>Highton, Patrick; University Hospitals of Leicester NHS Trust, NIHR Applied Research Collaboration East Midlands<br>Hadjiconstantinou, Michelle; University of Leicester, Diabetes Research Centre, College of Life Sciences<br>Pritchard, Rebecca; University Hospitals of Leicester NHS Trust, NIHR Leicester BRC; University of Edinburgh<br>Lock, Selina; University of Leicester, Library Research Services<br>Singh, Sally; University Hospitals of Leicester NHS Trust, 12. Department of Respiratory Medicine; Leicester Biomedical Research Unit, Centre for Exercise and Rehabilitation Science<br>Davies, Melanie; University of Leicester, Diabetes Research Centre; University Hospitals of Leicester NHS Trust, Leicester Diabetes Centre
**Primary Subject Heading**:	Rehabilitation medicine
Secondary Subject Heading:	Geriatric medicine
Keywords:	Rehabilitation medicine < INTERNAL MEDICINE, REHABILITATION MEDICINE, SPORTS MEDICINE

SCHOLARONE™
Manuscripts

Physical activity and sedentary behaviour interventions for people living with both frailty and multiple long-term conditions: a scoping review protocol

Hannah ML Young[1,2,3,], Thomas Yates[2], Paddy C Dempsey[2,4,5], Louisa Y Herring[1], Joseph Henson[1,2], Jack Sargeant[1,2], Ffion Curtis[6], Harini Sathanapally[6], Patrick Highton[1,6], Michelle Hadjiconstantinou[2], Rebecca Pritchard[1,7,9], Selina Lock[10], Sally J Singh[11,12], Melanie Davies[1,2]

1. Leicester Diabetes Centre, University of Hospitals of Leicester NHS Trust, Leicester, UK.
2. Diabetes Research Centre, College of Life Sciences, University of Leicester, UK.
3. Department of Respiratory Sciences, College of Life Sciences, University of Leicester, UK.
4. MRC Epidemiology Unit, Institute of Metabolic Science, University of Cambridge, Cambridge Biomedical Campus, Cambridge, UK.
5. Baker Heart and Diabetes Institute, Melbourne, Australia.
6. NIHR Applied Research Collaboration East Midlands, Leicester General Hospital, Leicester, UK.
7. University of Edinburgh, Edinburgh, UK
8. University Hospitals of Leicester NHS Trust, Leicester, UK
9. NIHR Leicester BRC, Cardiovascular Research Centre, Glenfield Hospital, Leicester, UK
10. Library Research Services, University of Leicester, Leicester, UK.
11. Centre for Exercise and Rehabilitation Science, Leicester Biomedical Research Unit, Leicester, UK.
12. Department of Respiratory Medicine, Glenfield Hospital, University Hospitals of Leicester NHS Trust, Leicester, UK.

Corresponding author:

Hannah Young

Leicester Diabetes Centre

Leicester General Hospital

Gwendolen Road

For peer review only - http://bmjopen.bmj.com/site/about/guidelines.xhtml

Leicester

UK

LE4 5PW

hy162@le.ac.uk

Abstract word count: 234

Main text word count: 3827

Keywords: multimorbidity, frailty, physical activity, exercise, review, multiple conditions, sedentary behaviour

For peer review only

Abstract

Introduction

The number of people living with multiple long-term conditions (MLTC) is predicted to rise. Within this population, those also living with frailty are particularly vulnerable to poor outcomes, including decreased function. Increased physical activity, including exercise, has the potential to improve function in those living with both MLTCs and frailty but to date, the focus has remained upon older people and may not reflect outcomes for the growing number younger people living with MLTC and frailty. For those with higher burdens of frailty and MLTCs, physical activity may be challenging. Tailoring physical activity in response to symptoms and periods of ill-health, involving family, and reducing sedentary behaviour may be important in this population. How the tailoring of interventions has been approached within existing studies is currently unclear. This scoping review aims to map the available evidence regarding these interventions in people living with both frailty and MLTCs.

Methods and analysis

We will use a six-stage process: (1) identifying the research questions; (2) identifying relevant studies (via database searches); (3) selecting studies; (4) charting the data; (5) collating and summarising and (6) stakeholder consultation. Studies will be critically appraised using the Mixed Methods Appraisal Tool.

Ethics and dissemination

All data in this project will be gathered through database searches. Stakeholder consultation will be undertaken with an established patient and public involvement group. We will disseminate our findings via social media, publication, and engagement meetings.

For peer review only - http://bmjopen.bmj.com/site/about/guidelines.xhtml

Strengths and limitations of this study

- We will identify primary studies that have incorporated sedentary behaviour, physical activity and/or exercise interventions and report how they were modified and tailored to the needs of those with multiple long-term conditions (MLTCs) and frailty.
- The design of the review has been shaped by strong patient and public involvement, and a diverse range of stakeholders will be involved throughout the review.
- The exclusion of non-English language studies may represent a limitation.
- There is potential that our review may miss some relevant studies, but an initial search indicates a need to maintain a balance between reviewing a feasible number of studies and ensuring all relevant articles are included.

For peer review only

Introduction

Physical inactivity and sedentary behaviour in people with multiple long-term conditions and frailty

The number of people living with multiple long-term conditions (MLTCs), defined as the coexistence of two or more chronic conditions, is a growing global health concern.[1] The presence of MLTC is associated with a myriad of poor outcomes, including low physical function and a resulting loss of independence and life participation.[2-9] Within this population, those also living with frailty, defined as "a multidimensional syndrome of decreased physiological reserve leading to increased vulnerability to minor health stressors", represent a group who are particularly vulnerable to these poor outcomes.[10 11] This is, in part, due to a bi-directional relationship between frailty and MLTCs.[12 13] Frailty appears to contribute to the onset of MLTCs, whilst the presence of MLTCs increases the risk of developing frailty.[14 15] Evidence suggests that people with MLTCs are at risk of becoming frail earlier.[16 17]

Sedentary behaviour (defined as any activity performed in a seated or reclined posture that requires low energy expenditure) and physical inactivity (insufficient amounts of moderate-to-vigorous physical activity) are associated with many long-term conditions.[18-20]Increasing physical activity and encouraging people to participate in exercise are often primary targets for intervention, but there is also growing interest in reducing sedentary behaviour.[21]Physical inactivity is common within the context of MLTCs. Approximately 29% of people living with MLTCs are unable to achieve physical activity recommendations of at 150 minutes of moderate physical activity per week, with levels of inactivity increasing with age.[22] In addition, there is an increased prevalence of probable sarcopenia in people with MLTCs.[23]Within this group there is a graded relationship between the degree of multimorbidity and sarcopenia, and an independent association between sarcopenia and physical inactivity.[24] People with MLTCs who can exercise demonstrate improved outcomes, including increased function, quality of life and life expectancy, indicating that physical activity and exercise may be important in the management of MLTCs.[21 22 25-29]

For peer review only - http://bmjopen.bmj.com/site/about/guidelines.xhtml

Sarcopenia, physical inactivity, and sedentary behaviour are also modifiable characteristics of the frailty phenotype and existing evidence suggests that undertaking resistance exercise and physical activity may delay both the onset and progression of frailty and improve outcomes such as physical function.[30-35] Increased physical activity is also a cornerstone of obesity management, which has also been associated with MLTCs and frailty.[36-38] Reducing sedentary behaviour and engaging in physical activity and exercise, particularly resistance training, across the life-course, and when living with MLTC, is now a major focus of clinical and public health policy.[21 32 39-41]

Compared to data from 2015, an 86% increase in the number of people living with two more conditions is predicted by 2035, in those aged over 65 years alone, making effective interventions for this groups increasingly important.[42 43] This is underscored by a recent Public Health England report which underlines the negative impact of the COVID-19 pandemic on sedentary behaviour and physical activity, particularly upon those with MLTCs who were advised to shield. Modelling predicts that the impact of deconditioning and increased falls risk will lead an estimated cost of £211million per year to health and social care services.[44]

Sedentary behaviour and physical activity and interventions

Existing systematic reviews indicate that structured exercise can lead to improvements in function, depression, and anxiety, but have mixed impacts upon quality of life in people living with MLTCs who are not frail.[45-47] Similarly, systematic reviews of exercise in people living with frailty suggest that those which include resistance training may improve function but have an unclear impact upon quality of life or ability to participate in activities of daily living.[48 49] Only one of these reviews, published in 2012, has examined the effectiveness of exercise for people living with both MLTCs and frailty, but focuses specifically on older people (aged over 60 years), and is limited by the interchangeable use of the concepts of disability and frailty.[45] This review indicated that exercise had a beneficial effect on mobility and physical functioning in this group, but not upon quality of life. Due to the range of intervention types included, the authors were unable to determine the most effective type of programme, and there was little discussion of any adaptations made, except for the phased approach in which

For peer review only - http://bmjopen.bmj.com/site/about/guidelines.xhtml

supervision was gradually reduced in one study. Contemporary research indicates that the demographics of people living with frailty and MLTCs are changing, with accelerated rates of both phenomena being observed in younger populations, people living with obesity, those with new and emerging health issues such as long-term HIV and early onset Type II diabetes.[15 36 50 51] An updated review of sedentary behaviour and physical activity interventions for those living with both MLTCs and frailty, that includes these groups is clearly warranted.[52]

Existing evidence suggests that identifying and targeting non-disease specific issues (for example symptoms such as pain, fatigue breathlessness) are important factors associated with increased effectiveness.[47 53] This may be important within the context of physical activity and exercise interventions, where uptake is hampered by concerns that symptoms may be aggravated.[54] Additionally, adherence in this population is low and the sustained engagement of people with both MLTCs and frailty is disrupted by periods of ill-health, exacerbation of their conditions and increased symptom burden, coupled with lack of guidance and support on safe and effective physical activity in these circumstances.[36 55-57]

Guidance on the management of people living with MLTCs and frailty highlights a need to identify and tailor treatment strategies.[41] Tailored adaptations to physical activity and exercise may encourage people living with frailty and MLTC to engage and support them to continue as much as they are safely able during periods of ill-health, increased symptomology and functional variability. Such strategies are urgently needed, as those who do complete combined resistance and aerobic exercise programmes appear to benefit from a range of improved outcomes, including improved exercise performance, physical activity levels and health status.[55] Whilst recently published guidance supports healthcare professionals to address concerns relating to the exacerbation of common symptoms at the point of initiating physical activity and exercise, they do not address how such interventions might need to be modified dependent upon symptom severity, which appears to be important for sustainability.[54] Even less is known about the role of interventions to address sedentary behaviour in this population.[20 53 58-60] Reducing sedentary behaviour may form a particularly useful adjunct approach for those who lack the capacity or motivation to undertake physical

For peer review only - http://bmjopen.bmj.com/site/about/guidelines.xhtml

activity and exercise. For these individuals, reducing and breaking up sedentary time may offer a more realistic, acceptable starting point, and a more sustainable segue into exercise.[61 62]

Finally, contextual factors, such as family involvement have been cited as key factors which may influence engagement and consequently outcomes.[47] Indeed, qualitative research indicates that family members and significant others are important ‘gatekeepers’ to health services and independence in people living with MLTCS and frailty.[58 63]This suggests that family involvement may be an important component of an intervention. Evidence also suggests that MLTCs may occur within families beyond parent/child dyads, particularly within people from minority ethnic backgrounds.[64-67] These are associated with increased sedentary behaviour, physical inactivity, and poor levels of function at the family level.[64-69] Therefore, in addition to improving the engagement of those living with MLTCS and frailty, the involvement of family members may help to reduce their risk of developing MLTCs or support them to manage existing LTCs. Indeed, the findings of a recent systematic review suggest that the engagement of carers in an exercise ‘dyad’ leads to improvements in both caregiver physical and psychosocial health.[70] The involvement of carers in sedentary behaviour and physical activity interventions is less clear. Caregiving requirements are likely to vary by age, and as a result of different clusters of MLTCs, together with frailty, so different caregivers and family members may need to be involved in different ways. Clarifying how carers and family members have been involved in sedentary behaviour, physical activity and exercise interventions for people with MLTCs and frailty may help identify further ways to tailor such interventions and leverage intergenerational support.

Overall, understanding the characteristics of sedentary behaviour, physical activity and exercise interventions for people living with both MLTCs and frailty is an important first step to identifying safe, scalable, and sustainable physical activity interventions which may be beneficial in this population.

For peer review only - http://bmjopen.bmj.com/site/about/guidelines.xhtml

Justification for this review

Scoping reviews seek to 'determine the body of literature on a given topic and give a clear indication of the volume of literature available, as well as an overview of its focus'.[71] Whilst there is emerging evidence in the contexts of MLTCs and frailty independently, less is known about the role of sedentary behaviour and physical activity interventions in those living with both. Specifically, we would like to identify primary studies in these conditions, including younger adults where very little research has been conducted. Conducting a scoping review will enable us to draw on the wider literature and begin to develop a programme theory to underpin our intervention design targeting this increasing population.[72-76] Of relevance to this population is scoping the range of ways in which interventions may be adapted and tailored. An approach that allows diverse evidence to be integrated will provide a more nuanced understanding of this, which may be lost within a systematic review.[76 77]

Aim and objectives

The aim of this review is to map the available evidence on the use of sedentary behaviour, physical activity and exercise interventions in people living with both frailty and MLTCs. The identified evidence will be used to highlight gaps within the existing literature, including a determination of whether there is sufficient evidence in this area to undertake a systematic review. The results of the review will be used to inform the design and development of an intervention for this population.[73]

Methods and analysis

Design

This scoping review will follow a six-stage process informed by guidance from Arksey and O'Malley (2005),[78] and subsequent refinements outlined by Levac (2010),[79] Colquhorn (2014)[80] and Daudt (2013).[81] This protocol follows the PRISMA reporting guidelines for scoping reviews.[82]

For peer review only - http://bmjopen.bmj.com/site/about/guidelines.xhtml

1.Identifying the research questions

To achieve the above aims the following questions will be addressed:

- What are the characteristics of sedentary behaviour, physical activity and exercise interventions that have been used with people living with both frailty and MLTCs?
- How have carers and relatives been included within the design, development, and delivery of these interventions?
- For each of the above, what approaches appear to contribute to improved engagement and outcomes, particularly physical function?

Patient and public representatives have been involved in shaping these questions from the outset. They described struggling to maintain physical activity levels during periods of ill-health, compounded by barriers to accessing existing services which could not accommodate their needs, or their variable ability to engage due to symptoms.

2. Identifying relevant studies

Inclusion and exclusion criteria

All relevant literature will be included, irrespective of the study design utilised. Studies will be included if the meet the following inclusion criteria (summarised within Table 1). These parameters may be refined and adapted if unmanageable volumes of eligible studies are identified following an initial search.[78]

For peer review only

For peer review only - http://bmjopen.bmj.com/site/about/guidelines.xhtml

Table 1. Inclusion and exclusion criteria

Inclusion criteria	Exclusion criteria
Any study design examining interventions or intervention content (e.g. single-group preintervention and postintervention, parallel- group design crossover or cluster, qualitative studies and process evaluations relating to interventions)	Studies in children or animals
Adults aged 18 and above	Presence of MLTCs not defined or < 50% of the sample report MLTCs
Living with both frailty and MLTCs	Recognised measure of frailty or validated proxy not used
Interventions with a sedentary behaviour, physical activity or exercise focus, including multicomponent interventions	Non-English language studies
Any setting	Studies of acute responses to sedentary behaviour, physical activity, or exercise, including interventions of less than one week in duration

For peer review only

Population

Adults aged 18 or above, living with both MLTCs and frailty. Within the review we define MLTCs as the co-existence of two or more chronic conditions (physical or mental) in a single individual.[1] Included long-term conditions, which could feasibly be influenced by physical activity, are outlined within Table 2. Studies where the presence of MLTCs could be assumed based solely upon participants' age or circumstances (e.g., resident of a nursing home) will be excluded.[53] However, given the relatively recent use of MLTCs as a term, we will include studies where the characteristics of the sample indicate that the majority (over 50%) are living

For peer review only - http://bmjopen.bmj.com/site/about/guidelines.xhtml

with MLTCs (e.g., Charlson co-morbidity index scores, counts of conditions). Similar approaches have been adopted within previous reviews of exercise and MLTCs.[46]

Table 2 Conditions included within the review

Type 2 diabetes	Asthma
COPD	Arthritis (osteo and rheumatoid)
Depression	Anxiety
Cancer (solid organ, haematological and metastatic)	HIV and AIDS
Chronic kidney disease	Chronic liver disease
Heart failure	Peripheral artery disease
Coronary artery disease	Hyperlipidaemia
Obesity	Ischaemic heart disease
Osteoporosis	Multiple Sclerosis
Parkinsons disease	

We define frailty as "a multidimensional syndrome of decreased physiological reserve leading to increased vulnerability to minor health stressors".[10] Studies using a validated frailty measure (e.g., Fried Frailty Phenotype, Clinical Frailty Scale, Frailty Index, Electronic Frailty Index) will be prioritised. Considering that frailty in younger people with MLTCs has only recently begun to be recognised, we will also include studies using proxy indicators of frailty (outlined within Table 3).[17 83-88] Studies which use other measures and cite supporting evidence to suggest association with frailty will also be included. Accepting that these instruments have only moderate specificity for the identification of frailty and have yet to be validated within younger frail populations, they may be more appropriate to, and more commonly used in, younger groups.[17 85] Studies including participants described as frail with no quantitative measurement of this will be excluded.

For peer review only - http://bmjopen.bmj.com/site/about/guidelines.xhtml

Table 3. Functional measures which are recognised proxy measures of frailty, and published cut-offs indicative of frailty.

Function test	Published cut points for the identification of frailty
Modified Physical Performance Test (PPT)	Score range: 0-36:<br>• Not frail: 32-36<br>• Mild frailty: 25-32<br>• Moderate frailty: 17-24<br>• Dependent: <17
Balance Performance Oriented Mobility Assessment (BPOMA)	BPOMA>19
Short Physical Performance Battery (SPPB)	A score of ≤ 7 is indicative of frailty
Timed Get-Up-and-Go test (TUAG)	A score of ≥ 9 seconds
Gait speed test	A gait speed of 0.8m/s is indicative of frailty<br>Taking more than 5 seconds to walk 4m
Sit to stand tests:<br>• Sit to stand 10 seconds<br>• Sit to stand 30 seconds<br>• Sit to stand 60 seconds<br>• Sit to stand 5 repetitions<br>• Sit to stand 10 repetitions	Dependent upon the type of sit-stand test used:<br>• ≥ 10 seconds for the five times sit stand<br>• Lower than published criterion standards to maintain independence, stratified by age and gender for other forms of the test
Handgrip strength	Scores within the lowest quartile, stratified by sex
SARC- F questionnaire	Score of ≥ 4

Studies which target only carers will be included, providing they are delivering unpaid care for people with the above inclusion criteria and the interventions focus upon sedentary behaviour, physical activity or exercise. Carer involvement is not included as a specific inclusion criterion, as we do not want to exclude relevant studies which did not include carers.

For peer review only - http://bmjopen.bmj.com/site/about/guidelines.xhtml

Concept

We define physical activity as "people moving, acting and performing within culturally specific spaces and contexts, and influenced by a unique array of interests, emotions, ideas, instructions and relationships".[89] Within this, we include exercise interventions as 'planned, structured, and repetitive bodily movement with the objective of improving or maintaining physical fitness'[90]. Multicomponent interventions, including rehabilitation programmes, will also be eligible, provided they meet the other inclusion criteria described. We will also include interventions which target sedentary behaviour, as previously defined.[18] Studies examining the acute effects of physical activity or sedentary behaviour interventions are excluded, as we are interested in the characteristics of programmes which are designed to influence longer-term outcomes such as physical function. An intervention duration of one week distinguishes acute interventions from non-acute behavioural interventions in free-living conditions.[91]

Context/settings

Studies from all settings will be eligible, including those in community, workplace, residential and hospital settings.

Searches will be limited to the year 2000 onwards to ensure they are relevant to current practice, and because the term 'frailty' as a medical condition of increased vulnerability is not well recognised prior to this.[92] Non-English language studies will be excluded. Any study design examining interventions or intervention content will be eligible for inclusion. The reference lists of relevant reviews will be used to identify other eligible papers not already included within the review. This will allow us to exclude papers which do not meet the eligibility criteria, and to avoid overstating the results of results of papers already included a primary sources.

For peer review only - http://bmjopen.bmj.com/site/about/guidelines.xhtml

Information sources

We will search for studies using the following databases and trials registries:

- For systematic reviews: Cochrane; PROSPERO; Database of Abstracts of Reviews of Effects (DARE).
- For published research: MEDLINE; EMBASE; Cumulative Index to Nursing and Allied Health (CINAHL); Web of Science; Sports Discus; PsycINFO; Pedro; Allied and Complementary Medicine (AMED); Cochrane Central Register of Controlled Trials (CENTRAL); Scopus.
- For grey literature: internet searching (e.g., Google Scholar), BIOSIS previews, Open Grey and the Index to Scientific and Technical Proceedings.

Relevant ongoing clinical trials will be included if they provide sufficient information. The following databases will be searched for ongoing trials: CENTRAL; US National Institutes of Health Ongoing Trials Register (ClinicalTrials.gov); and the World Health Organization International Clinical Trials Registry Platform (ICTRP).

Meeting abstracts will not be included as they are unlikely to include the level of information required. Additional relevant literature will be identified via handsearching the references of included papers, drawing on forward and backwards citation tracking and electronic "cited by" searches using Google Scholar. Where included trials reference linked protocol papers or qualitative research, these will be included. Searches will be updated prior to publication.

Searches

Search terms used are outlined in Appendix 1. This strategy has been developed by a health information specialist and the review team for MEDLINE and will be translated for other databases.

For peer review only - http://bmjopen.bmj.com/site/about/guidelines.xhtml

Searches will be executed by HMLY with support from the information specialist where required. Initially MEDLINE (PubMed) and CINAHL will be searched to pilot the search strategy. Key words related to the aims of the review from the titles and abstract of retrieved papers, and of the index terms used to describe the articles will be reviewed. The research team will then meet to discuss any refinements before further searches are conducted.[79] An initial search for terms relating to frailty /function OR terms relating multimorbidity AND terms relating to physical activity/ sedentary behaviour yielded an unmanageable amount of data and, therefore the search was adjusted to frailty/ function terms AND multimorbidity terms AND physical activity/ sedentary behaviour terms.

3.Selection of evidence

Following de-duplication, the remaining studies will be independently screened for eligibility by two reviewers, using the inclusion criteria outlined above. A systematic approach will be facilitated by reference and review management software (EndNote V.X9 and Covidence).[79 81] Firstly, titles and abstracts will be screened to remove ineligible records. Following this, at least two reviewers will screen the remaining full-text copies. At this stage demographic and clinical characteristics of the studies will be assessed to determine if they meet the criteria for MLTCs. Any discrepancies will be resolved through discussion and the inclusion of an additional reviewer if required. Authors will be contacted if, after full-text screening, it is still unclear whether to include/exclude an article. Authors will be contacted via email, with a further follow-up email two weeks later. The review team will meet regularly throughout each stage.

4.Data charting

A standardised data charting form will be used to comprehensively extract data from the included studies. The form will be developed by the review team based on the key objectives of the review. The form will be piloted by two reviewers on five studies to ensure all relevant information is extracted. Any changes to the form, and the rationale for these, will be recorded.

For peer review only - http://bmjopen.bmj.com/site/about/guidelines.xhtml

Microsoft Excel will be used to manage the extracted data. Where key information is missing from the full texts, authors will be contacted for additional information. The extraction of intervention characteristics, including how they are tailored and adapted will be guided by the template for intervention description and replication (TIDieR) checklist and the Consensus on Exercise Reporting Template (CERT).[93 94] For data relating to study methodology, relevant reporting guidelines will be used to guide the data extracted for each study type.[76] Key data to be extracted is outlined within Table 4. This list is not exhaustive, and data extracted may be subject to refinement.[76]

For peer review only - http://bmjopen.bmj.com/site/about/guidelines.xhtml

Table 4. Key data to be extracted within the review.

**Study details**	Author(s)
	Type of publication
	Year of publication
	Country of origin
**Description of methodology**	Aims/purpose
	Study design
	Inclusion and exclusion criteria
	Definition of frailty and frailty assessment or proxy functional measure used
	Primary and secondary outcomes
	Where applicable, definition of the carers involved
	Setting/ context (geographical, cultural, social environment and the organisational and political systems in which an intervention occurs)
	Sample size
	Characteristics of the study population, including:<br>Ethnicity<br>• Age<br>• Sex<br>• Indicators of socioeconomic status<br>• Presence of cognitive impairment<br>• Number, type and severity of long-term conditions<br>• Level of frailty of participants
	Where applicable, characteristics of carers or family members or significant others, including relationship to care receiver
**Description of intervention**	Focus of the intervention (sedentary behaviour, physical activity, exercise or combination)
	If applicable, the type of physical activity/ exercise, including equipment used and an outline of the components included
	The methods used to prescribe of the intervention
	The decision rules for determining the starting level

For peer review only

For peer review only - http://bmjopen.bmj.com/site/about/guidelines.xhtml

	The intervention duration and dose i.e. the prescribed frequency and intensity, the duration of the intervention and any maintenance period
	The mode of delivery (face to face, virtual, individual or group)
	The decision rules for determining progression
	Details of how the program was progressed and how this was monitored
	The location/ setting of delivery (e.g., home-based or in-centre) including any necessary infrastructure or other relevant features
	Details, methods of, and reasons for tailoring, personalisation, or adaptation.
	Details and methods of any modifications to the intervention during the study, particularly in relation to periods of ill-health and fluctuating symptomology
	Intervention rationale, programme theory, or goals
	The physical or informational materials used in the intervention, including those provided to participants or used in intervention delivery or in training of intervention providers
	The procedures, activities, and/or processes used in the intervention, including any enabling or supportive activities, motivation strategies used (e.g. counselling/education; environmental modification; prompting; self-monitoring; social comparison; financial incentives)
	Amount of supervision, including contact time
	The intervention providers, including their qualifications/expertise, background and any training provided to them
	Description of how carers, relatives or significant others are included within the design, development, or delivery of the intervention
	How intervention fidelity was assessed, and by whom, including methods for measuring adherence will also be included
	Strategies used to maintain or improve fidelity
**delivered**	How well the intervention was delivered as planned, including recorded levels of adherence to the programme

For peer review only - http://bmjopen.bmj.com/site/about/guidelines.xhtml

To differentiate between physical activity and sedentary behaviour interventions, we will use the approach described by Hadgraft et al.[91] Finally, we will record the outcomes used to determine the interventions effectiveness, including outcomes relating to carer health and wellbeing, and the effects of the intervention on physical function and other relevant outcomes (particularly physical activity, sedentary behaviours and measures of quality of life). Key findings that relate to the scoping review questions will also be recorded alongside an assessment of the quality of the study.

Critical appraisal

Critical appraisal of the included studies will provide information on the quality of the available evidence. The identification of lower quality research will strengthen the identification of gaps within the existing literature.[79] Studies will not be excluded based on quality.

We will assess the quality of the included studies using the using the Mixed Methods Appraisal Tool (MMAT) which has previously been used in scoping reviews.[95-97] The MMAT is a brief, but reliable, critical appraisal tool which provides a single method for assessing the quality of a range of qualitative, quantitative and mixed-methods study designs.[76 98 99] Two reviewers will be familiarised with the tool and devise a strategy for applying the tool in a systematic manner.[98 99] Following this, they will undertake the assessment independently, with recourse to additional members of the team where required.[99] A detailed presentation of the ratings of each included criterion will be reported.[100]

5.Synthesis and reporting

We plan to use a convergent synthesis design, based upon each of the research questions identified.[101] Qualitative and quantitative data will be summarised using a narrative approach, supplemented by descriptive statistics, tables and figures as appropriate. Following this, qualitative and quantitative data relating to each question will be integrated using mixed-methods joint displays.[101] We anticipate that joint displays will be organised according to the

For peer review only - http://bmjopen.bmj.com/site/about/guidelines.xhtml

domains outlined within the TIDieR and CERT checklists.[93 94] Within the joint displays, consideration will also be given to the effectiveness of the identified interventions and the quality of the studies. This will enable us to better identify those interventions or components which appear to lead to more favourable outcomes, and to assess areas of ongoing uncertainty. The results of this review will be reported in accordance with PRISMA guidance.[82]This proposed synthesis plan will be further refined toward the end of the review.[76]

6.Stakeholder consultation

The final stage will involve discussing the review results with two stakeholder groups. The first will comprise approximately six to eight people living with both frailty and at least two co-existing long-term conditions from those included within this review, alongside with their carers/family members. These individuals are already engaged as members of a patient and public involvement (PPI) group for a study related to this review. We have already taken steps to ensure that we are actively considering equality diversity and inclusion and will continue to ensure the composition of this group is broadly representative of the population of interest.

The second group will comprise approximately six to eight exercise and healthcare professionals, and researchers with expertise in intervention development. These individuals are already engaged as collaborators for a study related to this review or will be approached via existing links with local hospital and community health and research networks. We will include broad representation from professionals and academics with an interest in the management of MLTC and frailty alongside expertise in the speciality areas of interest in the review (respiratory, musculoskeletal, mental health, metabolic and infectious diseases, cardiology, neurology oncology and geriatrics).

The two groups will meet separately to mitigate any issues relating to power differentials, and to allow both groups to discuss their views openly. A single meeting for each group will be

For peer review only - http://bmjopen.bmj.com/site/about/guidelines.xhtml

facilitated by HMLY. Meetings will not be audio-recorded but a co-facilitator will take notes and observe group interactions. Meetings will be held face-to-face or virtually as circumstances allow. Given the heavy burden of healthcare and the increased potential for periods of illness, where lay stakeholders cannot attend, they will be consulted individually. Face to face meetings will be held in accessible locations. Materials will be adapted for the needs of those with sensory impairments as required. Lay members will be rewarded and recognised for their time and expertise in accordance with current guidance.[102]

The objective of the meetings will be to understand stakeholders' perspectives regarding any potential evidence informed interventions identified.[73] We will present a summary of our findings to inform discussions. They will be asked to consider how well any identified interventions fit the proposed context, and where and how they may need further adaptation.[74] Areas where there are ongoing uncertainties will be flagged. Stakeholders will also be asked for their views on areas which have not been addressed within the current evidence base which may be important to them. Following the meetings, a plan for future development and/or adaptation work will be developed and shared with the whole group for further comment. Feedback from the stakeholder consultation will be integrated with the findings of the review and described in the final report.

Patient and public involvement

In addition to shaping the research questions, PPI group members have provided feedback on how to make stakeholder meetings inclusive and accessible. They have also advised on the dissemination strategy adopted.

Ethics and dissemination

All data in this project will be gathered through database searches. Ethical approval to publish information from the stakeholder consultation process will be sought from the University of Leicester Internal Review Board for the stakeholder consultation stage of this review.

For peer review only - http://bmjopen.bmj.com/site/about/guidelines.xhtml

We will disseminate our findings at relevant academic and clinical meetings and by publishing them in an academic journal within the field. Results will also be disseminated to people living with both frailty and MLTC, and carers via the Lifestyle and Cardiovascular Biomedical Research Units involvement forum, relevant local and national patient charities, and our social media platforms.

Summary

The numbers of people living with MLTC are predicted to rise, and within this population those also living with frailty are particularly vulnerable to poor outcomes such as decreased function and independence. Sedentary behaviour, physical activity and exercise interventions form an integral part of chronic disease and frailty management and may also be important for those living with both MLTC and frailty. Existing systematic reviews suggest that exercise has the potential to improve function in those living with both MLTCs and frailty, but to date the focus has remained upon older people, which may not reflect those living with MLTC, who become frail at a younger age. Tailoring the intervention in response to symptoms and periods of ill-health and involving family members appears to be important in this population, but how this has been approached within existing studies is currently unknown. Additionally, the role of broader interventions which address sedentary behaviour are unclear. This scoping review aims to map the available evidence on the use of sedentary behaviour, physical activity and exercise interventions in people living with frailty and MLTCs. The results will inform the design of a tailored intervention and highlight gaps, directing future research.

Authors' contributions

HMLY conceived the study idea. HMLY, PD, LH, JS, JH, FC, HS, PH, SL, MH, RP, SS, TY, MD designed the protocol, HMLY, MH and RB devised the stakeholder collaboration process, HMLY and SL designed the search strategy. HMLY prepared the manuscript. PD, LH, JS, JH, FC, HS, PH, SL, MH, RP, SS, TY, MD reviewed final manuscript. Mentorship was provided by TY, SS, MD. HMLY, PD, LH, JS, JH, FC, HS, PH, SL, MH, RP, SS, TY, MD contributed important intellectual

For peer review only - http://bmjopen.bmj.com/site/about/guidelines.xhtml

content during manuscript drafting or revision. HMLY accepts accountability for the overall work ensuring that questions pertaining to the accuracy or integrity of any portion of the work are appropriately investigated and resolved.

Funding statement

The research was funded by the National Institute for Health Research (NIHR) Leicester Biomedical Research Centre, Collaboration for Leadership in Applied Health Research and Care East Midlands (CLAHRC EM). HMLY is supported by grants from the NIHR (NIHR301593). SJS. is supported by the Collaboration for Leadership in Applied Health Research and Care East Midlands. The views expressed in this publication are those of the authors and not necessarily those of the NHS, the NIHR or the Department of Health and Social Care. The funders had no role in the study design; collection, analysis, and interpretation of the data; writing the report; and the decision to submit the report for publication.

Competing interests' statement

All authors have no conflicts of interest to declare.

References

1. The Academy of Medical Sciences . Multimorbidity: a priority for global health research. 2018 [Available from: https://acmedsci.ac.uk/file-download/82222577 accessed 20th December 2021.
2. Williams JS, Egede LE. The association between multimorbidity and quality of life, health status and functional disability. *The American journal of the medical sciences* 2016;352(1):45-52.
3. Jacob ME, Ni P, Driver J, et al. Burden and Patterns of Multi-morbidity: Impact on Disablement in Older Adults. *Am J Phys Med Rehabil* 2020;99(5):359.
4. Marengoni A, Angleman S, Fratiglioni L. Prevalence of disability according to multimorbidity and disease clustering: a population-based study. *J* 2011;1(1):11-18.
5. Nicholson K, Griffith LE, Sohel N, et al. Examining early and late onset of multimorbidity in the Canadian Longitudinal Study on Aging. *J Am Geriatr Soc* 2021;69(6):1579-91.

For peer review only - http://bmjopen.bmj.com/site/about/guidelines.xhtml

6. Quiñones AR, Markwardt S, Botoseneanu A. Multimorbidity combinations and disability in older adults. *Journals of Gerontology Series A: Biomedical Sciences and Medical Sciences* 2016;71(6):823-30.
7. Read JR, Sharpe L, Modini M, et al. Multimorbidity and depression: a systematic review and meta-analysis. *J Affect Disord* 2017;221:36-46.
8. Stafford M SA, Thorlby R, Fisher R, Turton C, Deeny S. . Briefing: Understanding the health care needs of people with multiple health conditions 2017 [Available from: file:///C:/Users/Hannah/Downloads/Understanding%20the%20health%20care%20needs%20of%20people%20with%20multiple%20health%20conditions%20(1).pdf accessed December 10th 2021.
9. Sibley KM, Voth J, Munce SE, et al. Chronic disease and falls in community-dwelling Canadians over 65 years old: a population-based study exploring associations with number and pattern of chronic conditions. *BMC geriatr* 2014;14(1):1-11.
10. Clegg A, Young J, Iliffe S, et al. Frailty in elderly people. *The lancet* 2013;381(9868):752-62.
11. Aarts S, Patel K, Garcia M, et al. Co-presence of multimorbidity and disability with frailty: an examination of heterogeneity in the frail older population. *J* 2015;4(3):131.
12. Villacampa-Fernandez P, Navarro-Pardo E, Tarin JJ, et al. Frailty and multimorbidity: Two related yet different concepts. *Maturitas* 2017;95:31-35.
13. Espinoza SE, Quiben M, Hazuda HP. Distinguishing comorbidity, disability, and frailty. *Curr Geriatr Rep* 2018;7(4):201-09.
14. Aprahamian I, Mamoni RL, Cervigne NK, et al. Design and protocol of the multimorbidity and mental health cohort study in frailty and aging (MiMiCS-FRAIL): unraveling the clinical and molecular associations between frailty, somatic disease burden and late life depression. *BMC psychiatry* 2020;20(1):1-11.
15. Hanlon P, Nicholl BI, Jani BD, et al. Frailty and pre-frailty in middle-aged and older adults and its association with multimorbidity and mortality: a prospective analysis of 493 737 UK Biobank participants. *The Lancet Public Health* 2018;3(7):e323-e32.
16. Whitty CJ, MacEwen C, Goddard A, et al. Rising to the challenge of multimorbidity: British Medical Journal Publishing Group, 2020.
17. Spiers GF, Kunonga TP, Hall A, et al. Measuring frailty in younger populations: a rapid review of evidence. *BMJ Open* 2021;11(3):e047051.
18. Tremblay MS, Aubert S, Barnes JD, et al. Sedentary behavior research network (SBRN)–terminology consensus project process and outcome. *Int J Behav Nutr Phys Act* 2017;14(1):1-17.
19. Lee W-C, Ory MG. The engagement in physical activity for middle-aged and older adults with multiple chronic conditions: findings from a community health assessment. *J Aging Res* 2013;2013
20. Dempsey PC, Biddle SJ, Buman MP, et al. New global guidelines on sedentary behaviour and health for adults: broadening the behavioural targets. *Int J Behav Nutr Phys Act* 2020;17(1):1-12.
21. Dempsey PC, Friedenreich CM, Leitzmann MF, et al. Global public health guidelines on physical activity and sedentary behavior for people living with chronic conditions: a call to action. *Journal of Physical Activity and Health* 2020;18(1):76-85.
22. Vancampfort D, Koyanagi A, Ward PB, et al. Chronic physical conditions, multimorbidity and physical activity across 46 low-and middle-income countries. *Int J Behav Nutr Phys Act* 2017;14(1):1-13.

For peer review only - http://bmjopen.bmj.com/site/about/guidelines.xhtml

23. Dodds RM, Granic A, Robinson SM, et al. Sarcopenia, long-term conditions, and multimorbidity: findings from UK Biobank participants. *J Cachexia Sarcopenia Muscle* 2020;11(1):62-68.
24. Hurst C, Murray JC, Granic A, et al. Long-term conditions, multimorbidity, lifestyle factors and change in grip strength over 9 years of follow-up: Findings from 44,315 UK biobank participants. *Age and ageing* 2021;50(6):2222-29.
25. Cassidy S, Fuller H, Chau J, et al. Accelerometer-derived physical activity in those with cardio-metabolic disease compared to healthy adults: a UK Biobank study of 52,556 participants. *Acta Diabetol* 2018;55(9):975-79.
26. Steeves JA, Shiroma EJ, Conger SA, et al. Physical activity patterns and multimorbidity burden of older adults with different levels of functional status: NHANES 2003–2006. *Disabil Health J* 2019;12(3):495-502.
27. Autenrieth CS, Kirchberger I, Heier M, et al. Physical activity is inversely associated with multimorbidity in elderly men: results from the KORA-Age Augsburg Study. *Prev Med* 2013;57(1):17-19.
28. Martinez-Gomez D, Guallar-Castillon P, Garcia-Esquinas E, et al.Physical activity and the effect of multimorbidity on all-cause mortality in older adults. Mayo Clin Proc; 2017. Elsevier.
29. Chudasama YV, Khunti KK, Zaccardi F, et al. Physical activity, multimorbidity, and life expectancy: a UK Biobank longitudinal study. *BMC Med* 2019;17(1):108.
30. Cruz-Jentoft AJ, Sayer AA. Sarcopenia. *The Lancet* 2019;393(10191):2636-46.
31. Fried LP, Tangen CM, Walston J, et al. Frailty in older adults: evidence for a phenotype. *The Journals of Gerontology Series A: Biological Sciences and Medical Sciences* 2001;56(3):M146-M57.
32.World Health Organisation. Global action plan on physical activity 2018–2030: more active people for a healthier world. , 2018.
33. Rogers NT, Marshall A, Roberts CH, et al. Physical activity and trajectories of frailty among older adults: Evidence from the English Longitudinal Study of Ageing. *PLoS ONE* 2017;12(2):e0170878.
34. Peterson MJ, Giuliani C, Morey MC, et al. Physical activity as a preventative factor for frailty: the health, aging, and body composition study. *The Journals of Gerontology: Series A* 2009;64(1):61-68.
35. da Silva Coqueiro R, de Queiroz BM, Oliveira DS, et al. Cross-sectional relationships between sedentary behavior and frailty in older adults. *The Journal of sports medicine and physical fitness* 2016;57(6):825-30.
36. Yuan L, Chang M, Wang J. Abdominal obesity, body mass index and the risk of frailty in community-dwelling older adults: a systematic review and meta-analysis. *Age and Ageing* 2021
37. Lu Y, Liu S, Qiao Y, et al. Waist-to-height ratio, waist circumference, body mass index, waist divided by height0. 5 and the risk of cardiometabolic multimorbidity: A national longitudinal cohort study. *Nutrition, Metabolism and Cardiovascular Diseases* 2021;31(9):2644-51.
38. Pollack LM, Wang M, Leung MYM, et al. Obesity-related multimorbidity and risk of cardiovascular disease in the middle-aged population in the United States. *Prev Med* 2020;139:106225.
39. Ford JC, Ford JA. Multimorbidity: will it stand the test of time? *Age and ageing* 2018;47(1):6-8.

For peer review only - http://bmjopen.bmj.com/site/about/guidelines.xhtml

40. National Institute for Health and Care Excellence. Dementia, disability and frailty in later life – mid life approaches to delay or prevent onset NICE guideline [NG16] 2015 [Available from: https://www.nice.org.uk/guidance/ng16 accessed 20th December 2021.
41. National Institute for Health and Care Excellence. Multimorbidity: clinical assessment and management NICE guideline [NG56], 2016 [Available from: https://www.nice.org.uk/guidance/ng56 accessed 20th December 2021.
42. Kingston A, Robinson L, Booth H, et al. Projections of multi-morbidity in the older population in England to 2035: estimates from the Population Ageing and Care Simulation (PACSim) model. *Age and ageing* 2018;47(3):374-80.
43. Frost R, Rait G, Wheatley A, et al. What works in managing complex conditions in older people in primary and community care? A state-of-the-art review. *Health Soc Care Community* 2020;28(6):1915-27.
44. Public Health England. Wider impacts of COVID-19 on physical activity, deconditioning and falls in older adults 2021 [Available from: https://assets.publishing.service.gov.uk/government/uploads/system/uploads/attachment_data/file/1010501/HEMT_Wider_Impacts_Falls.pdf accessed 29th October 2021.
45. De Vries N, Van Ravensberg C, Hobbelen J, et al. Effects of physical exercise therapy on mobility, physical functioning, physical activity and quality of life in community-dwelling older adults with impaired mobility, physical disability and/or multi-morbidity: a meta-analysis. *Ageing Res Rev* 2012;11(1):136-49.
46. Bricca A, Harris LK, Saracutu M, et al. Benefits and harms of exercise therapy in people with multimorbidity: a systematic review and meta-analysis of randomised controlled trials. *Ageing Res Rev* 2020:101166.
47. Crowe M, Jordan J, Burrell B, et al. Clinical effectiveness of transdiagnostic health management interventions for older people with multimorbidity: a quantitative systematic review. *J Adv Nurs* 2016;72(10):2315-29.
48. Talar K, Hernández-Belmonte A, Vetrovsky T, et al. Benefits of resistance training in early and late stages of frailty and sarcopenia: a systematic review and meta-Analysis of randomized controlled studies. *J* 2021;10(8):1630.
49. Campbell E, Petermann-Rocha F, Welsh P, et al. The effect of exercise on quality of life and activities of daily life in frail older adults: A systematic review of randomised control trials. *Exp Gerontol* 2021;147:111287.
50. Tuttle LJ, Bittel DC, Bittel AJ, et al. Early-onset physical frailty in adults with diabesity and peripheral neuropathy. *Canadian journal of diabetes* 2018;42(5):478-83.
51. Gebregziabher M, Ward RC, Taber DJ, et al. Ethnic and geographic variations in multimorbidty: evidence from three large cohorts. *Soc Sci Med* 2018;211:198-206.
52. Parker S, Corner L, Laing K, et al. Priorities for research in multiple conditions in later life (multi-morbidity): findings from a James Lind alliance priority setting partnership. *Age and ageing* 2019;48(3):401-06.
53. Smith SM, Wallace E, O'Dowd T, et al. Interventions for improving outcomes in patients with multimorbidity in primary care and community settings. *The Cochrane database of systematic reviews* 2021;1(1):CD006560-CD60. doi: 10.1002/14651858.CD006560.pub4

For peer review only - http://bmjopen.bmj.com/site/about/guidelines.xhtml

54. Reid H, Ridout AJ, Tomaz SA, et al. Benefits outweigh the risks: a consensus statement on the risks of physical activity for people living with long-term conditions. *BJSM online* 2021
55. Maddocks M, Kon SS, Canavan JL, et al. Physical frailty and pulmonary rehabilitation in COPD: a prospective cohort study. *Thorax* 2016;71(11):988-95.
56. Young HM, March DS, Highton PJ, et al. Exercise for people living with frailty and receiving haemodialysis: a mixed-methods randomised controlled feasibility study. *BMJ Open* 2020;10(11):e041227.
57. Blackstock FC, ZuWallack R, Nici L, et al. Why don't our patients with chronic obstructive pulmonary disease listen to us? The enigma of nonadherence. *Ann Am Thorac Soc* 2016;13(3):317-23.
58. Mañas A, del Pozo-Cruz B, García-García FJ, et al. Role of objectively measured sedentary behaviour in physical performance, frailty and mortality among older adults: A short systematic review. *Eur J Sport Sci* 2017;17(7):940-53.
59. Prince S, Saunders T, Gresty K, et al. A comparison of the effectiveness of physical activity and sedentary behaviour interventions in reducing sedentary time in adults: a systematic review and meta-analysis of controlled trials. *Obes Rev* 2014;15(11):905-19.
60. Kehler DS, Theou O. The impact of physical activity and sedentary behaviors on frailty levels. *Mech Ageing Dev* 2019;180:29-41.
61. Dogra S, Copeland JL, Altenburg TM, et al. Start with reducing sedentary behavior: A stepwise approach to physical activity counseling in clinical practice. *Patient Educ Couns* 2021
62. Lerma NL, Cho CC, Swartz AM, et al. Isotemporal substitution of sedentary behavior and physical activity on function. *Medicine and science in sports and exercise* 2018;50(4):792.
63. Young HML Goodliffe S, Eborall HC,et al. SP418 Frailty and falls: A qualitative study exploring the experiences of patients living with end-stage renal disease and haemodialysis. . *Nephrology Dialysis Transplantation* 2019;Jun 1;34
64. Ellis KR, Hecht HK, Young TL, et al. Peer Reviewed: Chronic Disease Among African American Families: A Systematic Scoping Review. *Prev Chronic Dis* 2020;17
65. Singh K, Patel SA, Biswas S, et al. Multimorbidity in South Asian adults: prevalence, risk factors and mortality. *J Public Health* 2019;41(1):80-89.
66. Polenick CA, Birditt KS, Turkelson A, et al. Chronic condition discordance and physical activity among midlife and older couples. *Health Psychol* 2021;40(1):11.
67. Polenick CA, Birditt KS, Turkelson A, et al. Individual-level and couple-level discordant chronic conditions: Longitudinal links to functional disability. *Ann Behav Med* 2020;54(7):455-69.
68. Gomes GAdO, Luchesi BM, Gratão ACM, et al. Prevalence of physical inactivity and associated factors among older caregivers of older adults. *J Aging Health* 2019;31(5):793-813.
69. Greaney ML, Kunicki ZJ, Drohan MM, et al. Self-reported changes in physical activity, sedentary behavior, and screen time among informal caregivers during the COVID-19 pandemic. *BMC Public Health* 2021;21(1):1-9.
70. Doyle KL, Toepfer M, Bradfield AF, et al. Systematic review of exercise for caregiver–care recipient dyads: What is best for spousal caregivers—exercising together or not at all? *The Gerontologist* 2021;61(6):e283-e301.

For peer review only - http://bmjopen.bmj.com/site/about/guidelines.xhtml

71. Munn Z, Peters MD, Stern C, et al. Systematic review or scoping review? Guidance for authors when choosing between a systematic or scoping review approach. *BMC Med Res Methodol* 2018;18(1):1-7.
72. O'Cathain A, Croot L, Duncan E, et al. Guidance on how to develop complex interventions to improve health and healthcare. *BMJ Open* 2019;9(8):e029954.
73. O'Cathain A, Croot L, Sworn K, et al. Taxonomy of approaches to developing interventions to improve health: a systematic methods overview. *Pilot Feasibility Stud* 2019;5(1):1-27.
74. Moore G, Campbell M, Copeland L, et al. Adapting interventions to new contexts—the ADAPT guidance. *Bmj* 2021;374
75. Skivington K, Matthews L, Simpson SA, et al. A new framework for developing and evaluating complex interventions: update of Medical Research Council guidance. *Bmj* 2021;374
76. Noyes J, Booth A, Moore G, et al. Synthesising quantitative and qualitative evidence to inform guidelines on complex interventions: clarifying the purposes, designs and outlining some methods. *BMJ global health* 2019;4(Suppl 1):e000893.
77. Petticrew M, Rehfuess E, Noyes J, et al. Synthesizing evidence on complex interventions: how meta-analytical, qualitative, and mixed-method approaches can contribute. *J Clin Epidemiol* 2013;66(11):1230-43.
78. Arksey H, O'Malley L. Scoping studies: towards a methodological framework. *International journal of social research methodology* 2005;8(1):19-32.
79. Levac D, Colquhoun H, O'Brien KK. Scoping studies: advancing the methodology. *Implementation science* 2010;5(1):1-9.
80. Colquhoun HL, Levac D, O'Brien KK, et al. Scoping reviews: time for clarity in definition, methods, and reporting. *J Clin Epidemiol* 2014;67(12):1291-94.
81. Daudt HM, van Mossel C, Scott SJ. Enhancing the scoping study methodology: a large, inter-professional team's experience with Arksey and O'Malley's framework. *BMC Med Res Methodol* 2013;13(1):1-9.
82. Tricco AC, Lillie E, Zarin W, et al. PRISMA extension for scoping reviews (PRISMA-ScR): checklist and explanation. *Ann Intern Med* 2018;169(7):467-73.
83. Bouillon K, Kivimaki M, Hamer M, et al. Measures of frailty in population-based studies: an overview. *BMC geriatr* 2013;13(1):1-11.
84. De Vries N, Staal J, Van Ravensberg C, et al. Outcome instruments to measure frailty: a systematic review. *Ageing Res Rev* 2011;10(1):104-14.
85. British Geriatrics Society. Fit for frailty 2014 [Available from: https://www.bgs.org.uk/resources/recognising-frailty accessed 17th November 2021.
86. Studenski S, Perera S, Patel K, et al. Gait speed and survival in older adults. *Jama* 2011;305(1):50-58.
87. Makizako H, Shimada H, Doi T, et al. Predictive cutoff values of the five-times sit-to-stand test and the timed "up & go" test for disability incidence in older people dwelling in the community. *Phys Ther* 2017;97(4):417-24.
88. Bahat G, Ozkok S, Kilic C, et al. SARC-F questionnaire detects frailty in older adults. *The journal of nutrition, health & aging* 2021;25(4):448-53.
89. Piggin J. What is physical activity? A holistic definition for teachers, researchers and policy makers. *Front Sports Act Living* 2020;2:72.

For peer review only - http://bmjopen.bmj.com/site/about/guidelines.xhtml

90. Caspersen CJ, Powell KE, Christenson GM. Physical activity, exercise, and physical fitness: definitions and distinctions for health-related research. *Public health reports* 1985;100(2):126.

91. Hadgraft NT, Winkler E, Climie RE, et al. Effects of sedentary behaviour interventions on biomarkers of cardiometabolic risk in adults: systematic review with meta-analyses. *BJSM online* 2021;55(3):144-54.

92. Rockwood K, Howlett SE. Fifteen years of progress in understanding frailty and health in aging: BioMed Central, 2018:1-4.

93. Hoffmann TC, Glasziou PP, Boutron I, et al. Better reporting of interventions: template for intervention description and replication (TIDieR) checklist and guide. *Bmj* 2014;348

94. Slade SC, Dionne CE, Underwood M, et al. Consensus on exercise reporting template (CERT): explanation and elaboration statement. *BJSM online* 2016;50(23):1428-37.

95. Puts MTE, Toubasi S, Andrew MK, et al. Interventions to prevent or reduce the level of frailty in community-dwelling older adults: a scoping review of the literature and international policies. *Age and ageing* 2017;46(3):383-92.

96. Pluye P, Gagnon M-P, Griffiths F, et al. A scoring system for appraising mixed methods research, and concomitantly appraising qualitative, quantitative and mixed methods primary studies in mixed studies reviews. *Int J Nurs Stud* 2009;46(4):529-46.

97. Pace R, Pluye P, Bartlett G, et al. Testing the reliability and efficiency of the pilot Mixed Methods Appraisal Tool (MMAT) for systematic mixed studies review. *Int J Nurs Stud* 2012;49(1):47-53.

98. Souto RQ, Khanassov V, Hong QN, et al. Systematic mixed studies reviews: updating results on the reliability and efficiency of the mixed methods appraisal tool. *Int J Nurs Stud* 2015;52(1):500-01.

99. Hong QN, Gonzalez-Reyes A, Pluye P. Improving the usefulness of a tool for appraising the quality of qualitative, quantitative and mixed methods studies, the Mixed Methods Appraisal Tool (MMAT). *J Eval Clin Pract* 2018;24(3):459-67.

100. Hong QN, Fàbregues S, Bartlett G, et al. The Mixed Methods Appraisal Tool (MMAT) version 2018 for information professionals and researchers. *Education for information* 2018;34(4):285-91.

101. Cresswell and Plano-Clark. Designing and Conducting Mixed Methods Research. 3rd Ed. ed. United States: Sage International 2017.

102. NIHR. Payment guidance for researchers and professionals 2020 [Available from: https://www.nihr.ac.uk/documents/payment-guidance-for-researchers-and-professionals/27392 accessed 23rd October 2021.

For peer review only - http://bmjopen.bmj.com/site/about/guidelines.xhtml

Appendix 1. MEDLINE Search Strategy

1	exp comorbidity/
2	Comorbid*.mp.
3	co-morbid*.mp.
4	exp multimorbidity/
5	Multimorbid*.mp.
6	multi-morbid*.mp.
7	multidisease*.mp.
8	multi-disease*.mp.
9	exp multiple chronic conditions/
10	“multiple chronic conditions”.mp.
11	poly-morbidit*.mp.
12	polymorbidit*.mp.
13	poly-patholog*.mp.
14	polypatholog*.mp.
15	pluripatholog*.mp.
16	pluri-patholog*.mp.
17	multipatholog*.mp.
18	multi-patholog*.mp.
19	multicondition*.mp.

For peer review only

20	multi-condition*.mp.
21	exp Syndemic/
22	syndemic.mp.
23	"multiple health".mp.
24	"multiple chronic".mp.
25	"multiple long-term".mp.
26	condition*.mp.
27	ill*.mp.
28	disorder*.mp.
29	disease*.mp.
30	23 or 24 or 25
31	26 or 27 or 28 or 29
32	30 and 31
33	exp Diabetes Mellitus, Type 2/
34	“diabetes type 2”.mp.
35	“diabetes type II”.mp.
36	“early onset diabetes”.mp.
37	(diabetes adj3 type 2).mp.
38	(diabetes adj2 type ii).mp.
39	(diabetes adj type two).mp.

For peer review only

40	T2DM.mp.
41	T2D.mp.
42	33 or 34 or 35 or 36 or 37 or 38 or 39 or 40 or 41
43	exp Hypertension/
44	exp Blood Pressure/
45	"Blood Pressure".mp.
46	hypertens*.mp.
47	43 or 44 or 45 or 46
48	exp Heart Diseases/
49	exp Heart Failure/
50	exp Cardiovascular Diseases/
51	exp Coronary Disease/
52	cardiac.mp.
53	heart.mp.
54	cardiovascular.mp.
55	coronary.mp.
56	52 or 53 or 54 or 55
57	31 and 56
58	48 or 49 or 50 or 51 or 57
59	exp Vascular Diseases/

For peer review only

60	exp Carotid Artery Diseases/
61	cerebrovascular.mp.
62	vascular.mp.
63	Carotoid*.mp.
64	Arter*.mp.
65	61 or 62 or 63 or 64
66	31 and 65
67	exp Cerebrovascular Disorders/
68	59 or 60 or 66 or 67
69	exp Asthma/
70	asthma*.mp.
71	69 or 70
72	exp Pulmonary Disease, Chronic Obstructive/
73	COPD.mp.
74	“chronic obstructive pulmonary disease”.mp.
75	72 or 73 or 74
76	exp Hyperlipidemias/
77	exp Hypercholesterolemia/
78	exp Hypertriglyceridemia/
79	hyperlipid*em*.mp.

For peer review only

80	hypercholesterol*emia$.mp.
81	hypertriglycerid*emia*.mp.
82	76 or 77 or 78 or 79 or 80 or 81
83	exp Arthritis/
84	exp Arthritis, Rheumatoid/
85	exp Osteoarthritis/
86	arthritis.mp.
87	"rheumatoid arthritis".mp.
88	osteoarthritis.mp.
89	83 or 84 or 85 or 86 or 87 or 88
90	exp Depression/
91	exp Anxiety/
92	depression.mp.
93	anxiety.mp.
94	90 or 91 or 92 or 93
95	exp Neoplasms/
96	malignan*.mp.
97	cancer.mp.
98	95 or 96 or 97
99	exp HIV/

For peer review only

100	exp Acquired Immunodeficiency Syndrome/
101	"acquired immunodeficiency syndrome".mp.
102	AIDS.mp.
103	HIV.mp.
104	99 or 100 or 101 or 102 or 103
105	exp Kidney Failure, Chronic/
106	exp Kidney Diseases/
107	exp Renal Insufficiency/
108	renal.mp.
109	kidney.mp.
110	108 or 109
111	31 and 110
112	105 or 106 or 107 or 111
113	exp Liver Diseases/
114	liver.mp.
115	31 and 114
116	113 or 115
117	exp Osteoporosis/
118	osteoporosis.mp.
119	117 or 118

For peer review only

120	exp Obesity/
121	obes*.mp.
122	120 or 121
123	exp Multiple Sclerosis/
124	multiple sclerosis.mp.
125	"MS".mp.
126	123 or 124 or 125
127	exp Parkinson Disease/
128	“parkinsons disease”.mp.
129	"PD".mp.
130	127 or 128 or 129
131	peripheral artery disease.mp.
132	exp Peripheral Arterial Disease/
133	131 or 132
134	co-occur*.mp.
135	cooccur*.mp.
136	concurrent.mp.
137	coexist*.mp.
138	co-exist*.mp.
139	134 or 135 or 136 or 137 or 138

For peer review only

140	31 and 139
141	1 or 2 or 3 or 4 or 5 or 6 or 7 or 8 or 9 or 10 or 11 or 12 or 13 or 14 or 15 or 16 or 17 or 18 or 19 or 20 or 21 or 22 or 32
142	42 or 47 or 58 or 68 or 71 or 75 or 82 or 89 or 94 or 98 or 104 or 112 or 116 or 119 or 122 or 126 or 130 or 133
143	140 and 142
144	141 or 143
145	exp Frail Elderly/
146	exp Frailty/
147	frail*.mp.
148	prefrail.mp.
149	pre-frail.mp.
150	"functionally-impaired".mp.
151	Physical function*.mp.
152	"Physical frail*".mp.
153	Debility.mp.
154	145 or 146 or 147 or 148 or 149 or 150 or 151 or 152 or 153
155	exp Exercise/
156	exp Physical Fitness/
157	exp Sedentary Behavior/

For peer review only

158	exp Rehabilitation/
159	exp Exercise Therapy/
160	exercis*.mp.
161	“physical activit*.mp.
162	physical fitness.mp.
163	sedentary behavio*r”.mp.
164	sedentary.mp.
165	rehabilitat*.mp.
166	“exercise therapy”.mp.
167	155 or 156 or 157 or 158 or 159 or 160 or 161 or 162 or 163 or 164 or 165 or 166
168	144 and 154 and 167
169	limit 168 to yr="2000 -Current"

For peer review only
